# Self-assembling peptides imaged by correlated liquid cell transmission electron microscopy and MALDI-imaging mass spectrometry

**DOI:** 10.1038/s41467-019-12660-1

**Published:** 2019-10-23

**Authors:** Mollie A. Touve, Andrea S. Carlini, Nathan C. Gianneschi

**Affiliations:** 1 Department of Chemistry, International Institute for Nanotechnology, Chemistry of Life Processes Institute, and Simpson Querrey Institute for BioNanotechnology, Northwestern University, Evanston, IL 60208 USA; 2 Department of Chemistry and Biochemistry, University of California San Diego, 9500 Gilman Drive, La Jolla, CA 92093 USA; 3 Department of Materials Science and Engineering, Northwestern University, Evanston, IL 60208 USA; 4 Department of Biomedical Engineering, Northwestern University, Evanston, IL 60208 USA

**Keywords:** Peptides, Self-assembly, Mass spectrometry, Transmission electron microscopy

## Abstract

We describe the observation of stimuli-induced peptide-based nanoscale assemblies by liquid cell transmission electron microscopy (LCTEM). LCTEM offers the opportunity to directly image nanoscale materials in liquid. Despite broad interest in characterizing biological phenomena, electron beam-induced damage remains a significant problem. Concurrently, methods for verifying chemical structure during or following an LCTEM experiment have been few, with key examples limited to electron diffraction or elemental analysis of crystalline materials; this strategy is not translatable to biopolymers observed in nature. In this proof-of-concept study, oligomeric peptides are biologically or chemically stimulated within the liquid cell in a TEM to assemble into nanostructures. The resulting materials are analyzed by MALDI-imaging mass spectrometry (MALDI-IMS) to verify their identity. This approach confirms whether higher-order assemblies observed by LCTEM consist of intact peptides, verifying that observations made during the in situ experiment are because of those same peptides and not aberrant electron beam damage effects.

## Abstract (web summary)

Peptide self-assembly into fibres is prevalent in nature in normal tissue and in degenerative diseases. Here, the authors report on the coupling of liquid cell TEM with MALDI-imaging mass spectrometry to study processes of peptide fibre formation, in solution, in real time with nanoscale resolution.

## Introduction

**Fig. 1:**
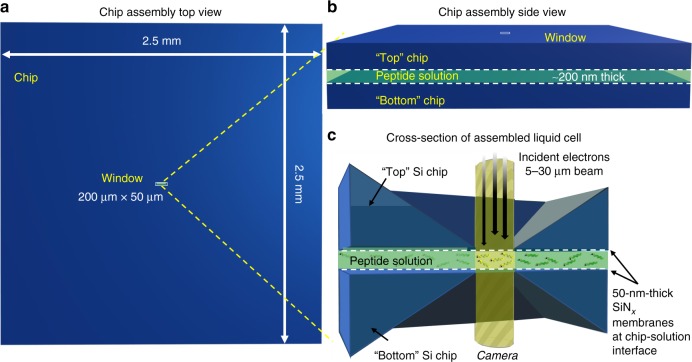
Liquid cell transmission electron microscopy (LCTEM) experimental setup. **a** Top view of a silicon nitride (SiN_*x*_) chip with an electron-transparent window of 200 µm× 50 µm. **b** Side view of two chips (top and bottom) assembled as a liquid cell containing a peptide sample solution, with a liquid thickness of ~200 nm. **c** Cross-section of an assembled liquid cell, placed in the tip of an LCTEM holder, where the windows of each chip are overlapping for imaging. The SiN_*x*_ film in this case is 50 nm thick

The direct imaging of biological materials and processes by liquid cell transmission electron microscopy (LCTEM) is of broad interest, with the potential to impact our understanding of nanoscience broadly, and nanoscale processes in biology in particular^1–3^. LCTEM employs a liquid cell, made in a number of different geometries with various materials^4–8^, including via sandwiching a thin (~<500 nm) film of liquid (e.g., water, organic solvents) between two silicon nitride (SiN_*x*_) chips (silicon chips, with SiN_*x*_ observation windows—50 µm × 200 µm), sealed at the tip of a TEM holder, and inserted into a side loading, standard microscope. The electron beam is then incident on the thin SiN_*x*_ window of the top chip, through the sample, exiting the cell via the bottom chip through an identical window (Fig. 1). The extent and type of electron beam damage occurring during any LCTEM experiment, on solvent and sample, is of critical importance, and for sensitive biological systems and materials, these effects have the potential to greatly perturb or alter processes of importance merely by imaging them^9^.

**Fig. 2:**
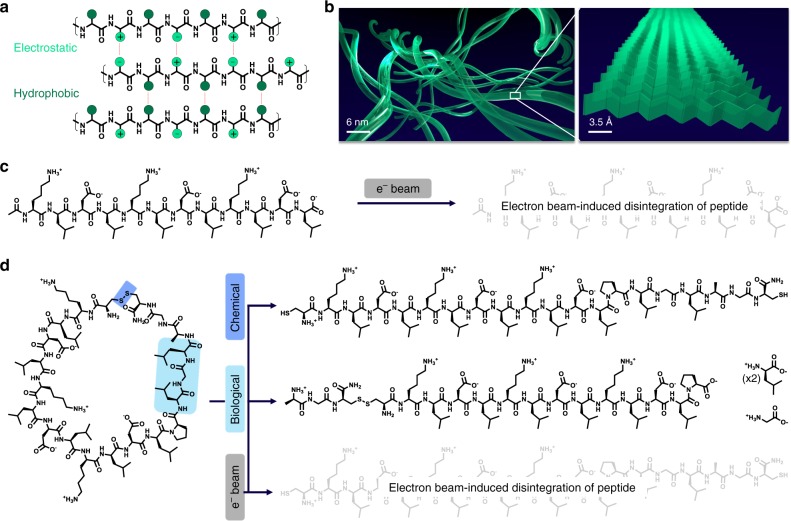
Stimuli-responsive peptides. **a** Self-assembly through electrostatic (light green) and hydrophobic (dark green) interactions between peptide strands. **b** Peptides stack as β-sheets that further assemble as fibrils. **c** KLDL peptide and hypothetical electron beam damage product. **d** cycKLDL with built-in stimuli-responsive sites and their products following chemical reduction of the disulfide bond, biological cleavage by enzymatic activity, and hypothetical electron beam damage product

One set of ambitious examples comes from work seeking to study live biological cells by LCTEM^2,10–14^. In this work, electron beam damage is clearly deleterious with negative effects, observed, for example, by way of cell shrinkage during imaging. Foundational studies are needed that show, in a robust fashion, whether even simple biological processes such as enzyme activity, or biomolecular recognition and assembly, can be imaged by LCTEM without severe beam-induced effects. Naturally occurring nanostructures and microstructures consist of assemblies of sugars, lipids, nucleic acids, peptides, and/or proteins among other minerals and metabolites. With a nearly infinite set of possible materials to study, an informative starting point for investigations into LCTEM electron beam damage mitigation and analysis for biomolecules would be peptides. Specifically, peptides/proteins that assemble into nanoscale fibrils, matrices, and gels are common motifs found in nature. Furthermore, relatively small peptides can be easily prepared and purified with precision by solid-phase synthesis, can be analyzed by sensitive mass spectrometry methods, and can be designed to assemble through noncovalent interactions into larger supramolecular structures. In turn, peptides can form soft matter assemblies through electrostatic and hydrophobic interactions providing a test case for establishing non-damaging electron flux and cumulative flux conditions for this broad class of beam-sensitive materials (Fig. 2). Two reported peptides, a linear self-assembling peptide (SAP) and a cyclic adduct that resists assembly as a dispersed progelator, could serve as similar yet distinct constructs for understanding how self-assembled and disperse systems are affected by irradiation during an LCTEM experiment^15^. Further, the cyclic progelator contains both a disulfide linkage and an enzyme-responsive moiety. When reduced or proteolytically cleaved, respectively, the cyclic peptide linearizes to form fibrils or hydrogels depending on whether concentrations are lower or higher, respectively^15^. Therefore, we aimed to establish electron flux and cumulative electron flux conditions, which are non-damaging to the peptides, and then apply these conditions to directly observe the linearization of the cyclic peptide to generate nanoscale architectures.

Several studies have utilized post-mortem analysis after LCTEM imaging for characterizing crystalline and/or inorganic materials, including in situ-generated metal-organic^16^ and covalent-organic^17^ framework structures, confirmed by electron diffraction, and nanoparticle composition identified via energy-dispersive X-ray spectroscopy^18^. To our knowledge, no techniques have been demonstrated for probing the chemistry of soft matter following an LCTEM experiment; this is a core unmet need in the development of this characterization technique. This type of post-mortem analysis is critical in any analysis of the components that lead to observations in terms of nanoscale assembly, disassembly, or morphology change/transition. It has been demonstrated that the electron flux and cumulative electron flux utilized during LCTEM experiments are key parameters to consider and to mitigate beam-induced structural and chemical damage to the materials being imaged, as well as radiolysis of the solvent itself^9,19,20^. Determining the degree of influence of the electron beam to a given system during in situ TEM experiments has largely been limited to observing qualitative changes to a material’s structure^11,21–23^, observing its disintegration^6,16^, or by intentionally growing a nanomaterial directly using electron beam effects^5,7,24–28^. A major source of the difficulty in assigning chemical structures and identities to materials following an LCTEM experiment arises as a result of the small quantity of sample present in the low volumes found within a liquid cell (~<3 pL). This means that it is difficult to apply standard analytical techniques, such as nuclear magnetic resonance (NMR) and solution or gas-phase mass spectrometry methods, that would provide confirmation of molecular structure. Matrix-assisted laser desorption ionization imaging mass spectrometry (MALDI-IMS) is an analytical technique largely used for probing the spatial chemistries of tissue specimens by raster scanning a small-diameter laser probe (<100 µm) across the surface of a sample in a predefined array to ionize the sample and generate a mass spectrum at each measurement point. This technique has been useful for detecting various drugs^29^, metabolites^30^, and biomolecules, including peptides, proteins, and lipids in tissue specimens^31–33^. We reasoned that MALDI-IMS would have the capability to produce a two-dimensional (2D) map of chemical species on LCTEM chip surfaces, and specifically of the observation window itself (0.01 mm^2^). Indeed, LCTEM chips are reliably one time use, which is essentially contaminated with the sample.

**Fig. 3:**
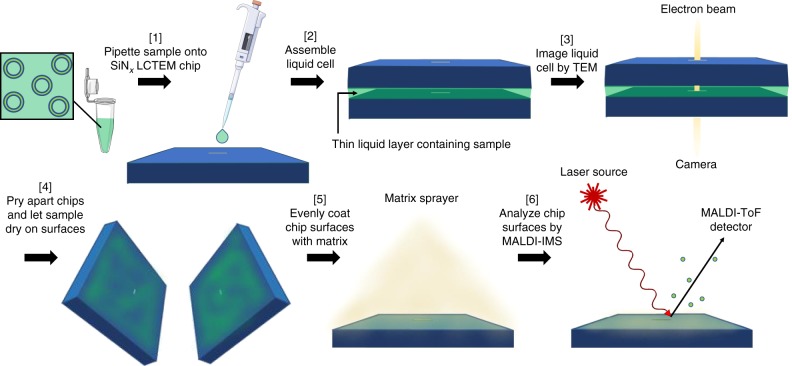
Overview of the workflow: LCTEM combined with MALDI-IMS post-mortem analysis. (1) The sample of interest is pipetted onto a bottom SiN_*x*_ LCTEM chip, and then (2) another LCTEM chip is placed on top and hermetically sealed. Next, (3) the assembled liquid cell is placed in a TEM for imaging. After imaging, (4) the chips are gently pried apart by hand and the sample is allowed to dry on chip surfaces. (5) Chip surfaces are evenly sprayed with matrix, and then (6) analyzed by MALDI-IMS. This figure was produced using Servier Medical Art

Herein, we report the use of MALDI-IMS as a sensitive post-mortem analytical technique, which possesses high spatial resolution and precision (Fig. 3). We show that MALDI-IMS allows for imaging of the observation windows (two are available: one from the top chip and one from the bottom chip), making direct use of the fact that the small sample volumes are not immediately amenable to extraction from that interface. Using this approach on each individual SiN_*x*_ chip, we collect on average 40,000 mass spectra, each of which contains ~90,000 data points averaged from 125 laser shots. This technique provides the opportunity to identify chemical species within mixtures, and to determine their individual relative intensities across a surface, with a spatial resolution defined by the MALDI laser focus diameter and raster width; both 50 µm in this case. We first made use of MALDI-IMS for determining the electron beam tolerance of two different peptides. Once tolerable imaging conditions were determined, dynamic LCTEM experiments involving the cyclic progelator peptide were performed, where assembly into nanoscale architectures was directly observed in response to a stimulus in situ. After these dynamic experiments, the chemistry of the peptide was directly probed and validated using MALDI-IMS.

## Results

### MALDI-IMS of analyte on SiN_*x*_ substrates

To assess whether MALDI-IMS can be used as a post-mortem analytical tool, we conducted preliminary experiments to first determine the feasibility of analyzing peptides by MALDI-IMS when the peptides are dried on a SiN_*x*_ LCTEM chip surface. Two different peptides were used in these studies: (a) KLDL, with the sequence Ac-KLDLKLDLKLDL-conh_2_, which self-assembles as a fibrous hydrogel; and (b) cycKLDL, with the sequence h_2_n-*CKLDLKLDLKLDLPLGLAGC*-conh_2_ (*indicates disulfide bond), the cyclic analog that is soluble until linearized by chemical reduction or enzymatic cleavage, and then cross-links into fibrous hydrogels (Fig. 2)^15^. KLDL was chosen as a standard for determining whether peptides retain their structure under low electron flux imaging conditions. Because cycKLDL forms structures of interest upon reduction or enzyme cleavage, it set the precedent for later studies on these peptide-based systems described below. In preliminary experiments, 2 µL of each peptide solution (0.5 mg mL^−1^) were pipetted onto SiN_*x*_ LCTEM chips and allowed to dry. The chips were mounted onto an indium-tin oxide (ITO) slide, and then spots of each peptide at various concentrations (0.01–1 mg mL^−1^) were spotted directly onto the ITO slide. The slide was evenly coated with α-cyano-4-hydroxycinnamic acid matrix, and then analyzed by MALDI-IMS using a 50 µm laser probe diameter (Supplementary Fig. 1). The expected mass spectra were acquired for each peptide (Supplementary Figs. 2–6). Indeed, signal for both KLDL and cycKLDL revealed intact peptide, with no signal crossover between different observed mass signals of KLDL [M + Na^+^]^+^ at 1491.31*m/z* (expected 1490.81 g mol^−1^), cycKLDL [M + H^+^]^+^ at 2136.72*m/z* (expected at 2139.67 g mol^−1^), and cycKLDL [M + H^+^ + 3Na^+^ + 3Cl^−^]^+^ at 2310.32*m/z* (expected 2314.99 g mol^−1^). This result sets the stage for appropriate analysis of mixtures of different peptide materials, as elucidated by 2D MALDI-IMS.

**Fig. 4:**
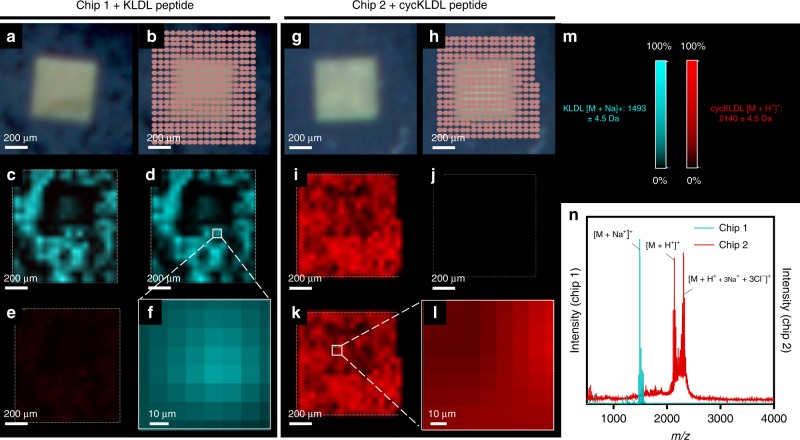
MALDI-IMS of LCTEM chips after assembly as a liquid cell containing KLDL or cycKLDL peptide. **a–f** Analysis of LCTEM chip with 10-nm-thick silicon nitride (SiN_*x*_) membrane window, coated with KLDL peptide. **a** Optical image of a square window of SiN_*x*_ LCTEM chip. **b** Map of laser spots with raster size of 50 µm. **c** Intensity map of merged KLDL and cycKLDL signal. **d** Intensity map of KLDL signal only. ROI for magnification in white box. **e** Intensity map of cycKLDL signal only. **f** Magnified ROI showing 2D map pixels. **g–l** Analysis of chip coated with cycKLDL peptide. **g** Optical image of square window of SiN_*x*_ LCTEM chip. **h** Map of laser spots with raster size of 50 µm. **i** Intensity map of merged KLDL and cycKLDL signal. **j** Intensity map of KLDL signal only. **k** Intensity map of cycKLDL signal only. ROI for magnification in white box. **l** Magnified ROI showing 2D map pixels. **m** Color intensity is displayed as 0–100% of total intensity on a logarithmic scale for each filter within the analyzed region. **n** Total averaged MALDI mass spectra from each chip plotted as absolute intensity vs. *m/z*. Filter (with manually defined range represented as ±) for KLDL [M + Na^+^]^+^ at 1493 ± 4.5*m/z* is shown in red. Filter for cycKLDL [M + H^+^]^+^ at 2140 ± 4.5 *m/z* is shown in blue. Intensity is calculated as integrated intensity for mass range. Source data are provided as a Source Data file

The next step was to probe the spatial resolution and sensitivity of MALDI-IMS when analyzing each peptide off LCTEM chip surfaces after liquid cell assembly. A liquid cell containing a 0.5 mg mL^−1^ peptide solution (KLDL or cycKLDL) was assembled, but not placed within the TEM for imaging, as no flux controls. Electron energy-loss spectroscopy (EELS) measurements estimated a 380–400 nm liquid layer thickness for a typical liquid cell assembly containing a 0.5 mg mL^−1^ cycKLDL solution (Supplementary Fig. 7). The assembled liquid cell remained on the bench top for 15 min, then the chips were gently pried apart by hand, dried, evenly coated with matrix, and analyzed by MALDI-IMS. Various techniques for chip separation were investigated to determine how separation may affect sample spreading and drying, but no differences were observed (Supplementary Fig. 4). Utilizing a 50 µm MALDI laser probe diameter, a mass spectrum was produced at each dwell position to generate a 2D intensity map of each chip surface (Fig. 4). For both the KLDL and cycKLDL peptides, strong signal intensities were observed across the surfaces of their respective chips at the expected *m/z* ratio, as shown by the application of *m/z* filters for each peptide.

### High electron flux conditions reveal damaged peptides

**Fig. 5:**
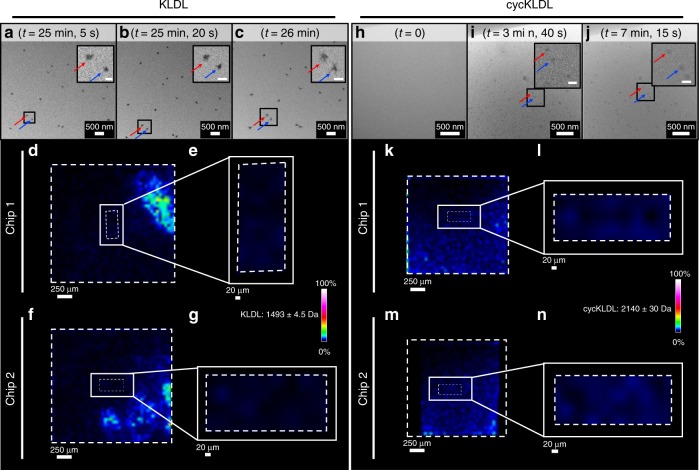
LCTEM of KLDL and cycKLDL under high flux conditions and post-mortem MALDI-IMS analysis. **a**–**c** LCTEM images acquired during irradiation of KLDL solution at high flux (26.6 e^−^ Å^−2^ s^−2^) for 30 min continuously. *t* = 0 corresponds to the start of imaging in the shown region of the liquid cell. Red and blue arrows distinguish particles tracked during the series of images. Scale bars of insets = 100 nm. **d** MALDI-IMS mapping of chip 1 from the LCTEM experiment shown in **a**–**c** with filter (with manually defined range represented as ±) for KLDL applied (1493 ± 4.5*m/z*). Boxes with dashed lines depict regions of interest analyzed by MALDI-IMS (entire chip surface or window only). **e** Zoom-in of MALDI-IMS mapping of the window region in **d** with filter for KLDL applied (1493 ± 4.5*m/z*). **f**, **g** Complimentary analysis as in **d**, **e** for chip 2. **h**–**j** LCTEM images acquired during irradiation of cycKLDL solution at high flux (20.4 e^−^ Å^−2^ s^−1^) for 30 min continuously, during which particle assembly and growth was observed. *t* = 0 corresponds to the start of imaging in the shown region of the liquid cell. Red and blue arrows in LCTEM micrographs distinguish particles tracked during the series of images. Scale bars of insets = 100 nm. **k** MALDI-IMS mapping of chip 1 from the LCTEM experiment shown in (**i**–**k**) with filter for KLDL applied (1493 ± 4.5*m/z*). Boxes with dashed lines depict regions of interest analyzed by MALDI-IMS (entire chip surface or window only). **l** Zoom-in of MALDI-IMS mapping of the window region shown in **k** with filter for KLDL applied (1493 ± 4.5*m/z*). **m**, **n** Complimentary analysis as in **k**, **l** for chip 2. Filter colors are displayed as 0–100% of integrated intensity on a logarithmic scale within the analyzed region

Next, we focused on imaging solutions of KLDL or cycKLDL by LCTEM under high flux conditions for 30 min continuously, with the purpose of damaging the peptides to eliminate the intact peptide MALDI-IMS signal within the irradiated window region of the liquid cell. Reports have shown that beam-induced effects on soft matter structures are minimized when imaging under low-flux conditions below ~1 e^−^ Å^−2^ s^−1^, while also limiting the cumulative flux^11,16,17,21^. For our studies, high flux values of 20–30 e^−^ Å^−2^ s^−1^ were utilized to damage the peptides. First, a liquid cell containing a solution of KLDL peptide at 0.5 mg mL^−1^ was prepared, and then the liquid cell holder was inserted into the microscope and imaged under high flux conditions (26.6 e^−^ Å^−2^ s^−1^) for 30 min continuously (cumulative flux = 47,900 e^−^ Å^−2^ s^−1^). Surprisingly, while imaging KLDL peptide under these conditions, many nanoscale, discrete, ill-defined, high-contrast particles were formed after 20 min of imaging within one region of the irradiated region and were observed to move rapidly, most likely an effect of charge accumulation of the SiN_*x*_ membranes and electric field production (Fig. 5, Supplementary Movie 1)^9,34^. When the beam was slowly relocated to surrounding regions of the liquid cell, these structures were initially absent, with similar structures forming upon irradiation. This suggests that localized imaging and radiolysis within one region of a liquid cell may not affect material and solvent compositions within other regions (>10 µm away) of the liquid cell (Supplementary Movie 2). After 30 min of imaging, the liquid cell holder was removed from the TEM, the chips were pried apart and the solution on the chips was dried. Analysis of the chips by MALDI-IMS showed a complete loss of intact KLDL signal (defined as 1493 ± 4.5*m/z* within the MALDI-IMS analysis) within the window region of the chips, where the electron beam was incident upon the liquid sample (Fig. 1c). We suspect that the observed structures during imaging were a result of aggregation of much smaller subunits of the original peptide, with masses small enough to blend in with background signals from the matrix^35^ in the MALDI-IMS analysis of the window regions (Supplementary Fig. 8).

Similarly, cycKLDL was imaged under high flux conditions (20.4 e^−^ Å^−2^ s^−1^) for 30 min continuously (cumulative flux 36,720 e^−^ Å^−2^ s^−1^) to induce damage to the irradiated peptide. Assembly into discrete, but well-defined and lower contrast spherical nanoparticles was observed within 60 s of imaging within one region of the liquid cell (Fig. 5h–j, Supplementary Fig. 9, Supplementary Movie 3). A few larger aggregates also appeared during imaging. MALDI-IMS of each chip revealed that chemical destruction of cycKLDL had occurred during imaging because the signal for the intact mass (defined as 2140 ± 30*m*/*z* within the MALDI-IMS analysis) was lost (Fig. 5k–n).

### Establishing non-damaging imaging conditions for peptides

**Fig. 6:**
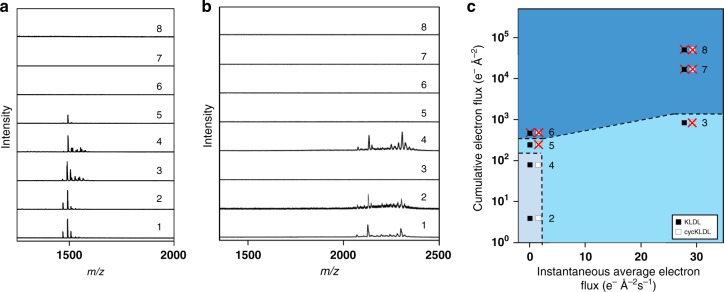
Establishing electron flux and cumulative electron flux thresholds for KLDL and cycKLDL. Compilation of **a** KLDL and **b** cycKLDL MALDI-IMS spectra acquired in window regions of LCTEM chips after imaging under: Condition **1** (no flux); **2** (low flux, pulsed, 30 min total); **3** (high flux, pulsed, 30 min total); **4** (low flux, 10 min continuous); **5** (low flux, 30 min continuous); **6** (low flux, 60 min continuous); **7** (high flux, 10 min continuous); and **8** (high flux, 30 min continuous). **c** Damage plot for peptides, comparing average electron flux and cumulative electron flux, with side-by-side square boxes indicating KLDL (black boxes) or cycKLDL (white boxes). Red *X* indicates that the MALDI signal for intact peptide was absent within the window region of the liquid cell as determined by MALDI-IMS. Source data are provided as a Source Data file

After establishing conditions under which the peptides are damaged by the electron beam of a TEM, the next step was to discern non-damaging conditions with sufficient signal-to-noise ratios. To do so, we systematically irradiated solutions of KLDL or cycKLDL peptide under various electron flux conditions for varying lengths of time (Supplementary Table 1, Supplementary Figs. 10–19). Electron flux values ranging from low flux (0.11 ± 0.07 e^−^ Å^−2^ s^−1^) to high flux (27.8 ± 5.7 e^−^ Å^−2^ s^−1^) were investigated. As it is difficult to reproduce absolute electron flux between individual experiments, we report the average flux values in Supplementary Table 1, with more emphasis on the magnitude of the electron flux. Under these different flux conditions, the solutions were imaged for varying lengths of time, from 10 min to 1 h, to probe the effect of cumulative flux on the soft matter samples. Imaging the peptide solutions at high flux for extended periods of time was expected to damage the materials, whereas the peptides were expected to remain intact when imaged under low-flux conditions for shorter total periods of time. Further, samples were also imaged under both low or high flux conditions in a pulsed manner, where the sample is exposed to the electron beam for a 1 s exposure at a time followed by blanking the beam for 59 s. This process was then repeated for a total of 30 min. The goal of the pulsed experiments was to capture periodic snapshots of the system while minimizing irradiation^17,21^. As in the control experiments, to determine sample integrity after imaging under a given set of imaging conditions, each liquid cell was gently pried apart, the sample on each chip was dried, and the chips were then mounted onto an ITO slide. Multiple sets of chips could be mounted on each ITO slide, as well as 2 µL spots of stock sample solutions as controls. The SiN_*x*_-coated surfaces of each chip were analyzed by MALDI-IMS to confirm the presence or absence of the intact peptide within the imaged observation window region of the liquid cell (Fig. 6).

The formation of artifactual nanostructures was avoided under all low-flux conditions (Supplementary Table 1) and when pulsed at high flux. Post-mortem MALDI-IMS confirmed intact KLDL peptide signal within the window regions of the liquid cells (Fig. 6a). Similar observations were made for cycKLDL (Fig. 6b), with two differences. First, when imaging under low-flux conditions for 30 min continuously (**5**), intact KLDL was detected by MALDI-IMS, whereas cycKLDL was not. Second, when imaged in the pulsed manner under high flux conditions (**3**), intact KLDL was detected by MALDI-IMS, but cycKLDL was not. Interestingly, through comparison of one peptide at similar cumulative electron flux (Fig. 6c), we observed that KLDL remained intact under high flux, pulsed conditions (**3**), but was degraded under low-flux conditions for 60 min of continuous imaging (**6**). These results reveal that peptide integrity is preferentially sensitive to electron flux.

### Observation of stimuli-induced peptide assembly by LCTEM

Having established that peptide solutions can be imaged without observable damage when performed under low-flux, pulsed conditions with minimal cumulative electron flux, we wanted to determine whether the assembly of cycKLDL into nanoscale architectures in response to a stimulus could be captured during an LCTEM experiment. To test this, we subjected the cycKLDL progelator, which resists self-assembly as a sterically controlled construct, to (1) disulfide reduction with tris(2-carboxyethyl)phosphine (TCEP) or (2) enzymatic cleavage with thermolysin. Importantly, these peptides are known to self-assemble into fibrous morphologies after linearization into SAPs^15^.

**Fig. 7:**
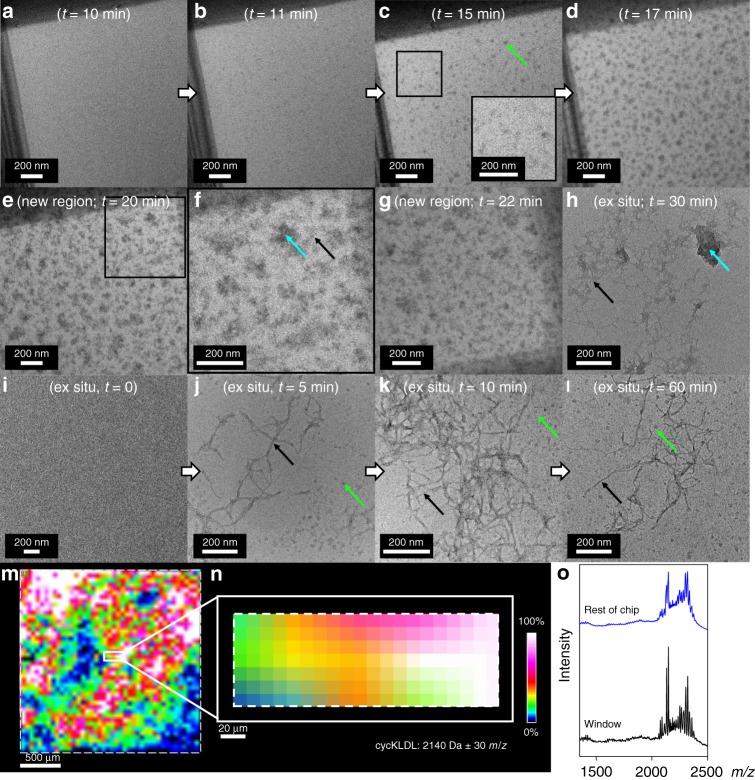
TCEP activation of cycKLDL to form nanoscale structures in situ and in bulk solution (ex situ). **a**–**d** LCTEM images of a solution of cycKLDL and TCEP acquired under low-flux, pulsed conditions, where *t* = 0 corresponds to initial mixing of cycKLDL and TCEP before liquid cell assembly. Inset in **c** shows zoom-in of boxed region. **e** Top right corner of the same liquid cell, which had not been previously irradiated. **f** Zoom-in of the region boxed in **e** depicting unstained architectures. **g** Bottom right corner of the same liquid cell, which had not been previously irradiated. **h** Dry-state image of uranyl acetate-stained architectures generated ex situ from the same reaction solution, which was imaged by LCTEM, with no agitation. **i**–**l** Uranyl acetate-stained, dry-state TEM images of cycKLDL and TCEP over a 60 min time period when formed ex situ in a standard test tube with agitation. Nanoparticle (green), aggregated node (blue), and fiber (black) architectures are denoted with colored arrows. **m** MALDI-IMS mapping of chip from the LCTEM experiment shown in **a**–**g** with filter (with manually defined range represented as ±) for cycKLDL applied (2140 ± 30*m/z*). **n** Zoom-in of MALDI-IMS mapping of the rectangular window shown in **m** with filter for cycKLDL applied (2140 ± 30*m/z*). **o** Averaged MALDI spectra of window region shown in **n** (black) and of the rest of the chip surface (blue). Filter colors are displayed as 0–100% of integrated intensity on a logarithmic scale within the analyzed region. Source data are provided as a Source Data file

We first utilized the disulfide bond in cycKLDL as a synthetic handle for chemical reduction by TCEP to induce assembly. A reductive solution of cycKLDL (1 mg mL^−1^) and TCEP was prepared outside the liquid cell, and then an aliquot (0.8 µL) of this solution was used to form a liquid cell, which was imaged immediately afterwards (~5 min from cell preparation). The sample was pulsed with the electron beam at 0.07 e^−^ Å^−2^ s^-1^ for a 1 s exposure to acquire an image, and then the beam was blanked for 59 s. This process was repeated for a total of 22 min (cumulative flux = 1.6 e^−^ Å^−2^ s^−1^). TCEP activation by LCTEM showed the appearance and growth of high-contrast, spherical nanoparticles starting at 11 min, where *t* = 0 corresponds to when the reaction mixture was initially mixed outside of the liquid cell (Fig. 7). These structures exhibited rapid initial growth accompanied by slowed growth up to 20 min (Fig. 7a–d). At ~20 min, a mixture of higher-contrast aggregated structures and fiber-like structures were observed in various regions of the liquid cell, which had not been irradiated previously (Fig. 7e–g). This indicates that the appearance of large structures during the in situ TCEP reduction is not dependent on the electron beam. A negative control, in which TCEP was inactivated at pH 3 prior to incubation with cycKLDL, was used to confirm that these structures were unable to form under similar imaging conditions (Supplementary Fig. 20). We note that the linear analog, KLDL, self-assembles into fibers in the range of pH 3.0–9.0. Therefore, the observed structures are formed through the reductive effect of active TCEP (Fig. 7). A separate reaction was set up in parallel (ex situ reaction) in a standard test tube where aliquots were taken at various time points. Dry-state TEM analysis of this ex situ reaction solution after 25 min of incubation at room temperature without agitation revealed a mixture of morphologies including fibers, aggregated nodes, spherical micelles, and globular structures (Fig. 7h, i–l). Notably, the aggregated nodes possessed much higher contrast than the other structures, which is the source of high-contrast structures in LCTEM (notably at 11–15 min). MALDI-IMS showed no change in mass spectra, which is expected as disulfide reduction results in an increase in peptide mass by only 2*m/z*. Importantly, the presence of a definite peptide signal within the window region by MALDI-IMS confirms that the TCEP-treated peptide was not damaged (Fig. 7m–o).

**Fig. 8:**
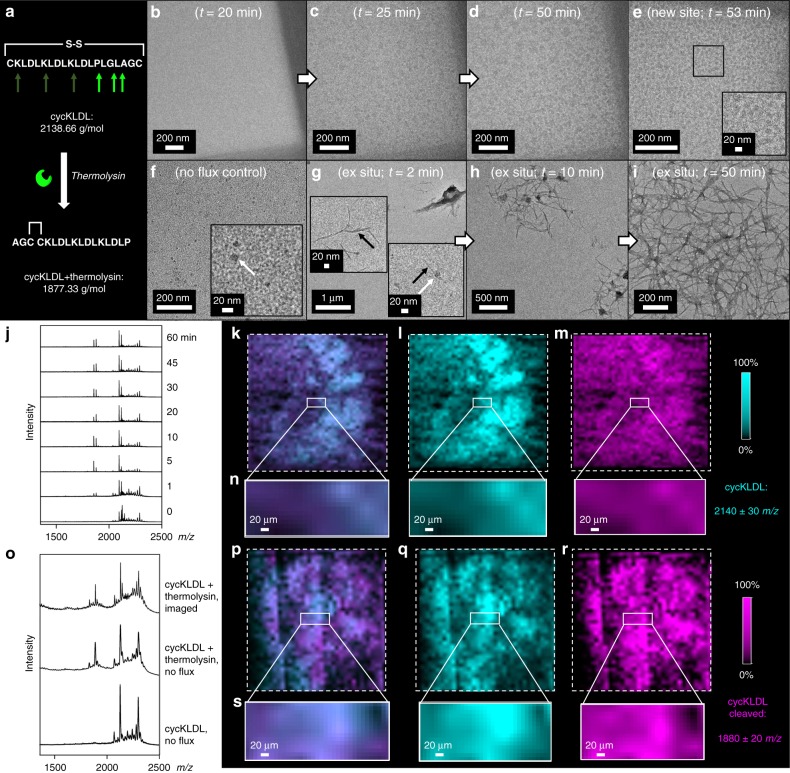
Enzyme activation of cycKLDL to form nanoscale structures. **a** Schematic of enzyme-induced linearization of cycKLDL by thermolysin. Observed cleavage locations indicated with bright green arrows. **b**–**d** LCTEM images of a solution of cycKLDL and thermolysin acquired under low-flux, pulsed conditions, where *t* = 0 corresponds to initial mixing of cycKLDL and thermolysin prior to liquid cell assembly. Images were acquired every 5 min. **e** Image of other corner of the same liquid cell shown in **b**–**d**, which had not been previously irradiated. **f** Dry-state image of particles on a SiN_*x*_ chip that was part of a liquid cell assembly that served as a no flux enzyme cleavage control experiment. White arrow denotes spherical nanoparticle. **g**–**i** Dry-state, uranyl acetate-stained TEM images of cycKLDL and thermolysin ex situ reaction mixture at various time points. White arrow denotes spherical nanoparticle. Black arrows denote fibers. **j** MALDI spectra of cycKLDL and thermolysin ex situ reaction mixture at various time points up to 60 min. **k**–**m** MALDI-IMS mapping of the surface of a chip from the LCTEM experiment shown in **b**–**d**, with different *m/z* filters applied. **n** Zoom-in of rectangular window regions of chips shown in **k**–**m** with applied *m/z* filters. **o** Averaged MALDI spectra of window region of an LCTEM chip, which had been assembled as a liquid cell containing cycKLDL with no flux (bottom), window region of an LCTEM chip, which had been assembled as a liquid cell containing cycKLDL and thermolysin solution and incubated for 1 h with no flux (middle), and window region of LCTEM chip containing cycKLDL and thermolysin solution after imaging by LCTEM under low-flux, pulsed conditions (top). **p**–**r** MALDI-IMS mapping of the surface of a chip that had been assembled into a liquid cell containing cycKLDL, but was not irradiated with the electron beam, as a no flux control. **s** Zoom-in of rectangular window regions of chips shown in **p**–**r** with applied *m/z* filters. Filter colors are displayed as 0–100% of integrated intensity on a logarithmic scale within the analyzed region. Source data are provided as a Source Data file

Next, we induced linearization of cycKLDL with thermolysin, a proteolytic enzyme, to generate assemblies (Fig. 8). The cleavage sites and expected masses for cycKLDL + thermolysin (1855.38*m/z* [M + H^+^]^+^ and 1877.18*m/z* [M + Na^+^]^+^) for linearized SAP after loss of Leu-Gly-Leu from h_2_n-*CKLDLKLDLKLDLP/LGL/AGC*conh_2_ (*disulfide bond; / indicated cut sites; underlined sequence is released) are provided in Fig. 8a. A solution of cycKLDL (1 mg mL^−1^) and thermolysin in water was prepared outside the liquid cell, and then an aliquot (0.8 µL) of this solution was immediately pipetted onto a bottom liquid cell chip. The liquid cell was assembled and immediately placed within the TEM for imaging. The beam was turned on at *t* = 14 min, where *t* = 0 was when the cycKLDL and thermolysin solution was initially mixed. During imaging, the sample was pulsed with the electron beam under low-flux conditions (0.04 e^−^ Å^−2^ s^−1^) for a 1 s exposure to acquire an image, and then the beam was blanked for 59 s. This process was repeated until noticeable assembly of spherical structures began to occur at *t* = 25 min (cumulative flux = 1 e^−^ Å^−2^ s^−1^) (Fig. 8b–f). After 25 min, the pulsed conditions were altered slightly so that a 1 s exposure was acquired only every 5 min. These structures continued to grow slowly with increasing contrast until *t* = 50 min, after which no changes were observed. The electron beam was relocated to other regions of the liquid cell to verify that similar structures had formed throughout the volume of the liquid cell, and not only within the irradiated region. Indeed, these spherical structures were found in previously unexposed regions, confirming that they were not artifacts of beam damage (Fig. 8e). As confirmation of spherical particle formation, a no flux control experiment was performed, wherein an identical liquid cell was made, containing cycKLDL and thermolysin, but the holder was not placed within the TEM during the reaction. After 1 h of incubation on a bench top, the liquid cell was gently pried apart and the solution on the chips was dried. The chips were then imaged in the dry state by TEM. Indeed, numerous 10–20 nm spherical particles were present across the surface of the chips (Fig. 8f). These diffusion-limited liquid cell reactions were compared to an identical reaction carried out in a vial, to see how morphology was affected by confinement (Fig. 8g–j). At early time points (e.g., 2 min), a mixture of short fibers and ~20 nm spherical nanoparticles were observed by dry-state TEM (Fig. 8g). At 10 min and on, longer fibers predominated and appeared to overlap with these nodes (Fig. 8h, i). These ex situ results show that thermolysin cleavage is detected by MALDI within 1 min (Fig. 8j). MALDI-IMS analysis of the chips from the in situ thermolysin cleavage experiment showed that the expected cleavage product was present across the surfaces of the LCTEM chips, including the imaged window regions of the chips, meaning the enzyme is active in the cell, and active during imaging (Fig. 8k–o). A strong signal for the intact cycKLDL peptide was also present across the chip surfaces (Fig. 8l). In both the in situ and ex situ experiments, the observed incomplete cleavage by MALDI (Fig. 8j, o) is likely influenced by low substrate concentrations and a lack of buffering salt. To more deeply probe why spherical nanoparticles were the dominating morphology observed within the LCTEM experiment, an additional no flux control was performed in a cell. After bench top incubation for 1 h, the liquid cell chips were pried apart and analyzed by MALDI-IMS to determine the extent of peptide cleavage. The expected cleavage products of 1855 and 1877*m/z* had formed and a significant amount of intact cycKLDL peptide was present (Fig. 8o–r). This demonstrates that enzyme cleavage and resulting product formation within a liquid cell environment is limited, as compared to when the reaction is performed in a standard test tube. This provides direct evidence that the different environments of the two types of reactions (reaction tube or liquid cell) may be the primary reason for the observed differences in self-assembly morphology^21,36^.

In a final control experiment for the proteolysis studies of cycKLDL, we imaged the peptide when mixed with inactivated thermolysin, for which peptide cleavage should not occur and thus assembly into nanoscale should not be observed. CycKLDL and inactivated thermolysin were mixed outside the liquid cell in a test tube, in the same manner as described above, and then 0.8 µL of this solution was immediately pipetted onto a bottom chip and a liquid cell prepared. The solution was imaged under low-flux, pulsed conditions (0.41 e^−^ Å^−2^ s^−1^) for 60 min. At no point was assembly observed (Supplementary Fig. 21). This confirms that active enzyme is necessary for proteolytic cleavage and subsequent self-assembly of cycKLDL into nanoscale structures observable by TEM.

## Discussion

We demonstrate the use of LCTEM for directly observing the formation of peptide-based assemblies in response to chemical reduction, and enzymatic cleavage. MALDI-IMS was shown to be a powerful technique for directly probing the chemistries of materials following an in situ TEM experiment and we propose this as a necessary form of post mortem analysis for soft matter, extendable to other systems from proteins and nucleic acids, to lipids and synthetic polymers. We demonstrated that various imaging conditions (electron flux and cumulative flux) can be easily probed to determine safe conditions for imaging beam-sensitive materials by employing MALDI-IMS, which correlated with TEM. For this proof-of-concept study, peptides were chosen as they can be prepared with absolute sequence control and have biological relevance. We reason that this correlated set of analytical methods can be applied to synthetic and biosynthetic polymers^37^, as increasingly complex questions arise in the fields of nanoengineering and materials science, stimulating the use of LCTEM. Ultimately, our aim is to expand these generalizable methods into buffered solutions comprising complex mixtures of active biomacromolecules, or mixtures of complex synthetic polymers capable of assembly into nanoscale and microscale soft matter structures.

In these experiments, cycKLDL demonstrated a higher beam sensitivity than its linear analog KLDL. When sterically constrained, cycKLDL does not assemble into networks (as occurs with KLDL), which likely increases individual peptide exposure to the electron beam. We suspect that KLDL possesses increased resistance to beam damage over that of cycKLDL due to its self-assembling nature^15^. In addition, because both peptides maintain integrity when imaged under low-flux, pulsed conditions, this suggests that allowing a system to equilibrate between electron beam pulses may help minimize beam-induced damage to the sample, and that cumulative electron flux is indeed a very important parameter to consider during LCTEM experiments.

Additionally, discrepancies between structure formation observed by LCTEM under high flux conditions and a lack of intact peptide signal by MALDI-IMS suggest that the formation of structures during an LCTEM experiment does not guarantee that the structures are self-assembled products of the imaged materials. Specifically, when either peptide was imaged under high flux conditions continuously for 30 min, structure formation was observed but intact peptide signal was completely absent by MALDI-IMS (Fig. 5). This proves the point that LCTEM can produce false impressions of nanomaterial formation; simply and obviously, care must be taken to provide evidence of the chemical species being observed as the elements forming observed assemblies. Extending LCTEM to the general scientific community interested in nanoscale materials will require these types of analyses to become routine parts of the workflow. Indeed, a broad understanding of the opportunities and limitations of any methodology are needed for widespread adoption.

In our studies with cycKLDL under minimal flux conditions, we had the opportunity to study self-assembly processes induced by both a chemical and biological stimulus. This made use of the engineered macrocyclic peptide structure with reductive disulfides and hydrolyzable substrate sequences, for which observable and analytically measurable changes occurred during treatment-induced linearization. In thermolysin experiments, numerous spherical nanoparticles formed in the liquid cell, whether the solution was imaged with the electron beam or not. Additionally, we provide further evidence of limited diffusion in a confined liquid cell as a potential source of deviation from assembly processes observed under standard, bulk reaction conditions^21^. Altogether, to our knowledge, these results constitute the first demonstration of enzymatic activity in LCTEM. This represents a step towards the study of increasingly biologically relevant systems by LCTEM.

Throughout these studies, we employed MALDI-IMS as a sensitive method for chemical analysis and mapping of LCTEM samples after in situ experiments that will only improve, with recent advances leading to MALDI-IMS instruments with spatial resolution of ~5 µm (Bruker Daltonics Rapiflex with Smartbeam 3D laser). For TEM in any imaging mode (e.g., cryogenic, dry state, environmental), the ease with which artifacts can be mistaken for materials of interest is always taken into account (e.g., staining artifacts and other sample preparation artifacts). LCTEM has added sources of artifacts, including how the electron beam influences nanomaterial motion in solution^9^, or adhere to the interface and interact with small molecules to grow, or disintegrate. Simply, because we are interested in dynamics by LCTEM, where changes in chemistry lead to changes in nanostructure morphology, tools for probing the chemistry must be employed routinely in tandem with the TEM experiment. This would be done together with comparison to other ex situ experiments performed in parallel including light scattering, and analysis by standard TEM, scanning transmission electron microscopy, and modeling studies. This study provides a route to chemical analyses of the very same samples imaged by LCTEM, identifying damaging and non-damaging imaging conditions. In summary, we establish a paradigm for observing complex biological interactions on the nanoscale and for elucidating their reaction products. Moreover, we anticipate MALDI-IMS for LCTEM will be generalizable to any material that yields to MALDI-MS.

## Methods

### General information

Amino acids used in Fmoc SPPS were purchased from AAPPTec and NovaBiochem. All other synthetic materials were obtained from Sigma-Aldrich and were used without further purification unless otherwise noted. Thermolysin (V4001) was acquired from Promega, as a lyophilized powder and resuspended at 1 mg mL^−1^ (27.6 µM) in milliQ H_2_O into 40 µL aliquots for storage at −80 °C.

### Peptide synthesis

Peptides were synthesized in an AAPPTec Focus XC peptide synthesizer. Peptides with C-terminal amides were synthesized on rink amide MBHA (4-methylbenzhydrylamine) resin and peptides with C-terminal carboxylic acids were synthesized on Wang-OH resin using double coupling conditions for the first amino acid. HBTU (*N*,*N*,*N*′,*N*′-tetramethyl-O-(1*H*-benzotriazol-1-yl) uronium hexafluorophosphate) was used as the general coupling agent. General peptide cleavage and deprotection was performed in 95:2.5:2.5 (%v/v) trifluoroacetic acid (TFA), triisopropyl silane, and H_2_O, respectively, for 2 h. Cleaved peptides were precipitated in cold anhydrous ether (3×) to yield solid crude. Semi-protected peptides containing Cys(acm) were purified prior to cyclization. To synthesize cyclic progelators, iodine was used to simultaneously deprotect Cys(acm) and initiate disulfide bond formation under dilute conditions to favor intramolecular macrocyclization. To a solution of semi-protected peptides (500 µM) in a mixture of acetic acid/methanol/H_2_O (1:16:4) was slowly added 0.1 M methanolic iodide until the yellow color persisted (~4–5 eq). The reaction was vigorously stirred at room temperature for 2 h and reaction completion was confirmed by liquid chromatography-mass spectrometry. After 2 h reaction, Amberlite IRA-400 resin (chloride form) was stirred in the solution for 1 h to quench excess iodine and absorb reacted iodide ions. Filtrate was placed on a rotary evaporator to remove acetic acid and methanol. The remaining solution was diluted with H_2_O and lyophilized to a white powder. By high-performance liquid chromatography (HPLC) no dimerization was observed.

### Peptide purification and analysis

Peptides were purified with a Jupiter Proteo90A Phenomenex column (2050 × 25.0 mm^2^) on an Armen Glider CPC preparatory phase HPLC over a 43 min gradient to yield 90–95% purity. The gradient solvent systems utilized Buffer A (H_2_O with 0.1% TFA) and Buffer B (acetonitrile (ACN) with 0.1% TFA). Crude peptide was prepared for purification in 5:25:70 acetic acid/Buffer A/Buffer B via initial dissolution in acetic acid with sonication, followed by the addition of ACN then H_2_O. Peptides were purified using a gradient of 25–45% Buffer B over 30 and 50 min for analytical HPLC and preparatory phase HPLC, respectively. Following purification, product identify was confirmed by electrospray ionization. HPLC purified peptides were dialyzed with 1 kDa MW cut-off tubing into milliQ H_2_O, sterile filtered through a 0.2 µm PES filter, and lyophilized to a powder.

### Dry-state TEM

TEM samples were prepared by depositing 4 μL aliquots of sample onto TEM grids (Formvar stabilized with carbon (5−10 nm) on 400 copper mesh, Ted Pella Inc.) that had previously been glow discharged in a PELCO easiGlow glow discharge unit for 30 s. The sample grid was lightly blotted with filter paper, and then stained with a 1% uranyl acetate solution and rinsed with water. Excess solution was removed by blotting the edge of the grid with filter paper, and then the grid was dried. Dry-state imaging was performed on a JEM-ARM300F (JEOL Ltd., Tokyo, Japan) operated at 300 keV. Micrographs were recorded on a 2k × 2k Gatan OneView-IS CCD camera (Gatan Inc., Pleasanton, CA, USA) using Gatan Digital Micrograph image acquisition software (Roper Technologies, Sarasota, FL).

### Liquid cell TEM

LCTEM chips with 50-nm-thick, 200 µm × 50 µm SiN_*x*_ membranes (Hummingbird Scientific, Lacey, WA, USA) were freshly glow discharged in a PELCO easiGlow glow discharge unit for 30 s. The lines of the LCTEM holder were filled with H_2_O prior to liquid cell assembly. Next, 0.8 μL of sample was pipetted manually onto the bottom chip, and then the liquid cell was assembled with the windows (50 µm × 200 µm) aligned in parallel, and the lines of the holder were sealed off without external flow. LCTEM imaging was performed in a JEM-ARM300F (JEOL Ltd., Tokyo, Japan) operated at 300 keV. Micrographs were recorded on a 2k × 2k Gatan OneView-IS CCD camera (Gatan Inc., Pleasanton, CA, USA) using Gatan Digital Micrograph image acquisition software (Roper Technologies, Sarasota, FL). The electron flux values used in LCTEM experiments were calculated using the beam current for each aperture selection, as measured by a Faraday Holder through vacuum, and the beam diameter incident upon the sample. For video acquisition, the TEM camera was operating in continuous imaging mode with a 0.5–1 s exposure time. Immediately following LCTEM experiments, the SiN_*x*_ chips were carefully separated and allowed to dry.

### Electron energy-loss spectroscopy

Scanning transmission electron microscopy (STEM) and EELS were performed using a JEOL ARM200CF STEM/TEM operated at 200 kV and gun emission set to 5 µA and equipped with a Gatan GIF-Quantum ER spectrometer. The beam convergence angle was set to 27.5 mrad. For EELS, a dispersion of 0.05 eV per channel and collection angle of 10 mrad was used. Simultaneously acquired ADF-STEM signal was collected within the scattering angle of 68–280 mrad. Data were processed using open-source software package HyperSpy^38^ and custom python codes. For each sample, measurements were taken at each corner of the window and averaged ± SD (*n* = 4).

### MALDI-IMS sample preparation

LCTEM chips were adhered with their SiN_*x*_ membranes facing upwards to the conductive face of an ITO- coated glass slide with 70–100 ohms resistivity (Bruker Daltonics), using ~0.5 µL nail polish and allowed to dry. To equalize the height difference from added SiN_*x*_ chips on the slide (~0.25 mm), five pieces of Scotch tape were applied to both short edges of the slide on the same side. As an internal quality control for confirming MALDI detector sensitivity, 3 × 2 µL spots of untreated peptide stock were deposited onto all slides. A 1% TFA solution (2 µL), used to acidify peptide cleavage products for improved sample ionization^39^, was carefully pipetted on top of chips and control spots to cover the entire surface and allowed to dry. Analysis with and without this acidification step showed no discernable difference in sample spreading across the chip surface. To each slide was sprayed an even thin-layer coating of HCCA (α-cyano-4-hydroxycinnamic acid) matrix (10 mg mL^−1^). Briefly, HCCA (10 mg mL^−1^) was dissolved in a mixture of ACN/water/acetone/TFA (48.0:46.8:5.0:0.2 v/v%) with an orbital shaker at room temperature for 1 h. Matrix (5 mL) was loaded into the injection loop of an HTX TM-Sprayer using a running buffer of 50:50% ACN/water operated with the HTX Imaging software. Matrix sample was flowed at 0.05 mL min^−1^ through a spray nozzle heated to 80 °C and sprayed under a constant N_2_ gas pressure of 10 Psi at 2 L min^−1^. Patterning was performed at a nozzle velocity of 1200 mm min^−1^, track spacing 3 mm, nozzle height 40 mm, and crisscross pattern over 8 total passes (2 s drying time per pass).

### MALDI-IMS analysis

Initial tests of MALDI-IMS on SiN_*x*_ chip surfaces were conducted using 10-nm-thick, standard (SiN_*x*_) window grids having 500 µm × 500 µm square SiN_*x*_ membrane windows (Norcada, Edmonton, AB, Canada). All following LCTEM/MALDI-IMS experiments were performed with chips having 50-nm-thick, 200 µm × 50 µm SiN_*x*_ membrane windows (Hummingbird Scientific, Lacey, WA, USA). Slides were mounted into an MTP Slide Adapter II and loaded onto a Bruker Autoflex III TOF MALDI mass spectrometer for analysis using the flexControl software (Bruker Daltonics 8237001). Samples were analyzed by MALDI-MS under reflector positive mode (500–4000 Da) using a 355 nm smartbeam 2 laser with a 50 µm focus diameter and 200 Hz frequency, a constant laser power of 25%, and a sum of 125 shots per spectrum. Spectra were collected within the region of 500–4000*m/z* using an accelerating voltage of 20 kV, and detector gain of 792 V. Region of interest (ROI) mapping and image analysis was performed in flexImaging software (Bruker Daltonics). Raw mass spectra within a giving ROI were averaged and the baseline subtracted. Final spectra in the main text are reported from a range of 1350–2000*m/z* for KLDL and 1350–2500*m/z* for cycKLDL to minimize background signal from the matrix^35^. Supplementary Figure 8 shows a sample spectrum where matrix background is observed up to ~1500*m/z*. For each 50 µm diameter pixel, integrated total signal generated from integrated mass spectra at each pixel within a defined mass range filter. All filters for KLDL used a mass range of 4.5*m/z*. To account for peak broadening from the presence of various ionic species, we expanded this range for cycKLDL in all figures after Fig. 3. Visual 2D maps were generated from these pixels and colorized according to 0–100% maximal signal on a logarithmic scale.

### Thermolysin cleavage of cycKLDL for LCTEM and MALDI-MS

To 200 µL of a stock solution of pre-sonicated (5 min) cycKLDL (1 mg mL^−1^ in H_2_O, pH 6.4) was added 40 µL of thermolysin stock at (26.7 µM in H_2_O) (1:85 enzyme/substrate ratio) at 21 °C. Samples were mixed for 10 s with gentle pipetting prior to analysis via LCTEM and separate time-course aliquot collections. To compare LCTEM results with that of enzymatic cleavage kinetics under no dose conditions, aliquots were collected at 0, 2, 5, 10, 20, 30, 45, and 60 min. Briefly, 2 µL aliquots were applied in duplicate to a MALDI slide and quickly dried over air to halt enzymatic activity. Concurrently, dry-state-stained TEM samples were prepared with 4 µL aliquots, as described above. To dry MALDI slide samples, 2 µL of HCCA matrix was applied and allowed to dry prior to MALDI-MS analysis. This experiment was conducted in duplicate.

### TCEP reduction of cycKLDL for LCTEM and MALDI-IMS

To a stock solution of pre-sonicated (5 min) cycKLDL (1 mg mL^−1^ in H_2_O, pH 6.4) was added 1.1 equivalents of TCEP with a final solution pH ~5. Samples were mixed for 10 s with gentle pipetting prior to analysis via LCTEM and separate time-course aliquot collections. To compare LCTEM results with that of TCEP reduction kinetics under no dose conditions, aliquots were collected at 0, 2, 5, 10, 20, 30, 45, and 60 min. Briefly, 2 µL aliquots were applied in duplicate to a MALDI slide and quickly dried over air to stop the reduction reaction. Concurrently, dry-state-stained TEM samples were prepared with 4 µL aliquots, as described above. To dry MALDI slide samples, 2 µL of HCCA matrix was applied and allowed to dry prior to MALDI-MS analysis. This experiment was conducted in duplicate. Control TCEP inactivation experiments utilized 3 equivalents of TCEP, whereby the pH of the solution dropped below 3.0, preventing disulfide reduction.

## Supplementary information

Supplementary Information

Supplementary Movie 1

Supplementary Movie 2

Supplementary Movie 3

Source Data

## Data Availability

The authors declare that all data supporting the findings of this study are available within the article and its Supplementary Information files or from the corresponding authors on reasonable request. The source data for all MALDI spectra in Figs. 4n, 6a, b, 7o, and 8j, o and Supplementary Figures 6, 7, 8, 10g, 11g, 12g, 13g, 14g, 15g 16g, 17g, 18g, and 19g are provided as a Source Data file.

## Code availability

Custom codes for EELS data analysis are available under request and are found at https://github.com/rmsreis/EELS-Liquid-Cell.
