# Supplementary material for: Self-assembling peptides imaged by correlated liquid cell transmission electron microscopy and MALDI-imaging mass spectrometry
Source: Nat Commun. 2019 Oct 23;10:4837. doi: 10.1038/s41467-019-12660-1 (PMC6811541; doi:10.1038/s41467-019-12660-1)
Supplement: Supplementary file 1 — Supplementary Information [file 41467_2019_12660_MOESM1_ESM.pdf]

Supplementary Information

Self-assembling peptides imaged by correlated liquid cell transmission electron microscopy and MALDI-imaging mass spectrometry

*M. A. Touve, et al. **

Supplementary Methods

Chip separation methods. To determine how chip separation affected sample spreading and drying on the chip surface, three methods for separation were investigated: (1) slide-off, where the two chips were gently slid apart laterally by hand; (2) razor blade, where the two chips were broken apart by inserting a razor blade between the two SiN_x surfaces; (3) freeze fracture, where tweezers were super-glued to either side of the attached chips, allowed to dry, then the chips were quickly plunged into liquid nitrogen to freeze the sample. After plunging, the chips were removed from liquid nitrogen and the tweezers were quickly opened to pry apart the chips. The razor blade and freeze fracture methods proved difficult to perform and did not result in any obvious differences in sample spreading as compared to the simpler slide-off method of separation.

Electron energy loss spectroscopy. EELS spectrum images of 20 by 20 pixels covering a region of 400 nm x 400 nm were taken from, both, empty and filled with 0.5 mg mL^{-1} cycKLDL in water, at each of the corners of the viewing membrane. These regions are a good representation of the location where the experiments were performed and the thinnest areas within the cell (**Supplementary Figure 7a, b**). Following Beer's law[39] which predicts an exponential decay of the unscattered incident beam, the average thickness of the cell was calculated in terms of the inelastic mean free path ($t\ \lambda^{-1}$) by determining the ratio of the number of unscattered electrons (I) in the zero-loss peak (ZLP) to the total number of incident electrons (I0). **Supplementary Figure 7c** shows low-loss EELS spectra from an empty and filled cells. It is clear that the spectrum from the filled cell has a more prominent plasmon peak and dominates the signal in the EELS spectrum. Typical values of optical gap (~7 eV) and plasmon energy 21 eV for water are observed. The average thickness was measured to be ~ 400 nm (with the thinnest point of ~380 nm) in the corner areas of the cell, where LCTEM imaging is usually conducted.

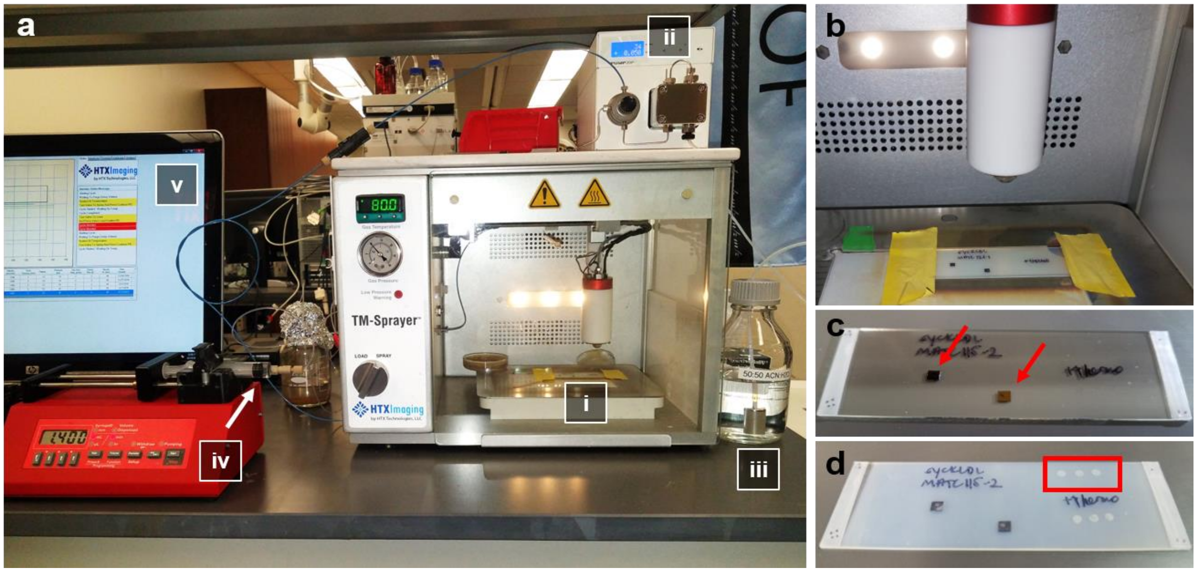

Supplementary Figure 1. MALDI-IMS slide matrix coating. a, Instrument setup includes (i) TM-Sprayer, (ii) solvent pump, (iii) running buffer, (iv) syringe pump with matrix sample (indicated with arrow), and (v) HTXImaging software. **b,** Magnified view of TM-Sprayer nozzle pattering over slide surface. **c-d,** Sample slide containing two chip surfaces from one experiment (c) before and (d) after matrix coating.

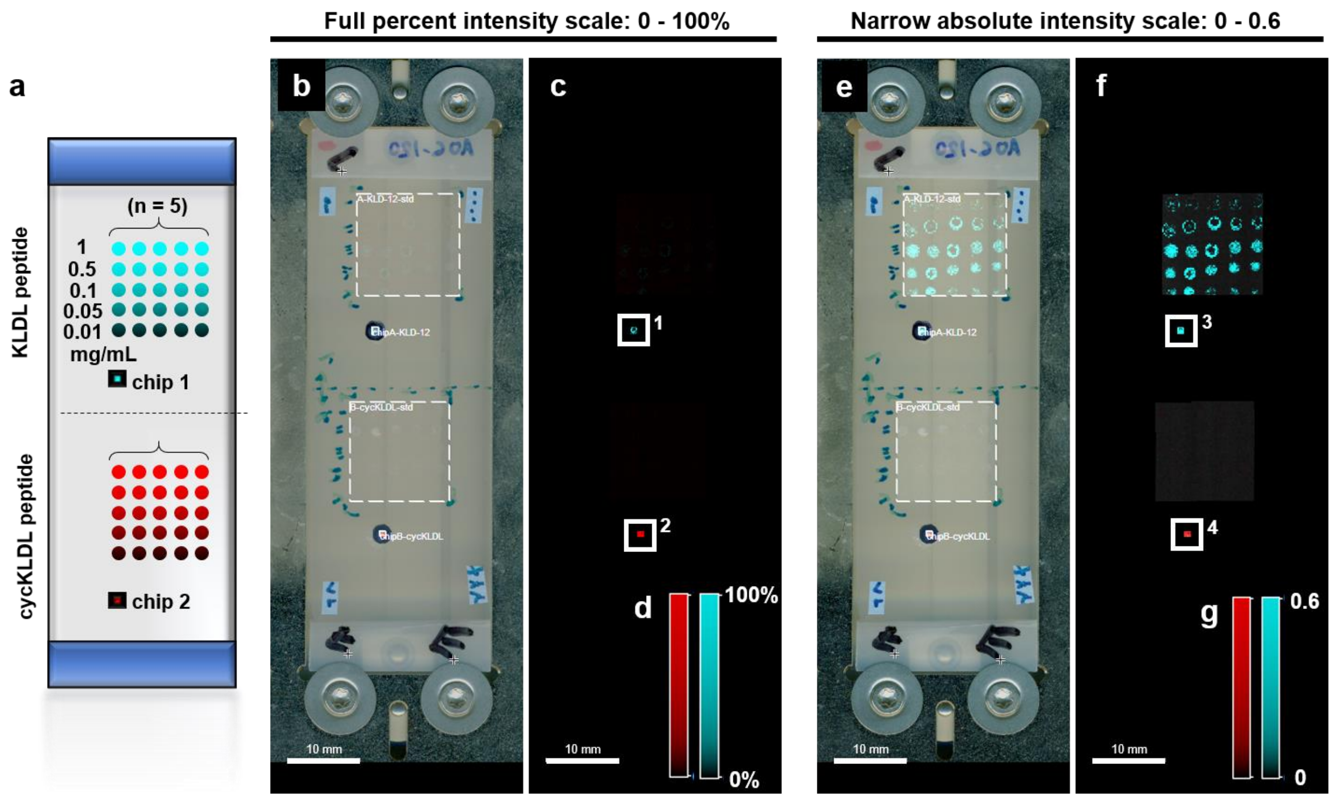

Supplementary Figure 2. Impact of Surface on MALDI Signal. Different thresholding reveals variable ionization potential of peptides on an ITO-coated glass slide (large regions) and silicon nitride LCTEM chips (small regions). a, Slide prep layout with KLDL peptide on the top half and cycKLDL peptide added to the bottom half. Gradient concentrations of 1, 0.5, 0.1, 0.05, and 0.01 mg mL^{-1} peptide were added as n = 5 spots per row on the ITO-coated glass slide surface (vol = 1.5 µL). Chips were coated with 0.5 mg mL^{-1} peptide (vol = 4 µL). b, Optical image of slide with merged signal from both peptide signals. c, Merged signal for both peptides only. White arrows indicate location of LCTEM chips. d, Color intensity is displayed as 0-100% of total intensity on a logarithmic scale for each filter within the analyzed region. e, Optical image of slide with merged signal from both peptide signals. f, Merged signal for both peptides only. White boxes indicate location of LCTEM chips. g, Color intensity is displayed as 0-0.6 absolute intensity on a logarithmic scale for each filter within the analyzed region. Filter (with manually defined range represented as ±) for KLDL $[M+Na^+]^+$ at 1493 ± 4.5 *m/z* is shown in blue. Filter for cycKLDL $[M+H^+]^+$ at 2137.5 ± 4 *m/z* is shown in red. Filter colors are displayed as 0-100% of integrated intensity on a logarithmic scale within the analyzed region. Scale bar = 10 mm.

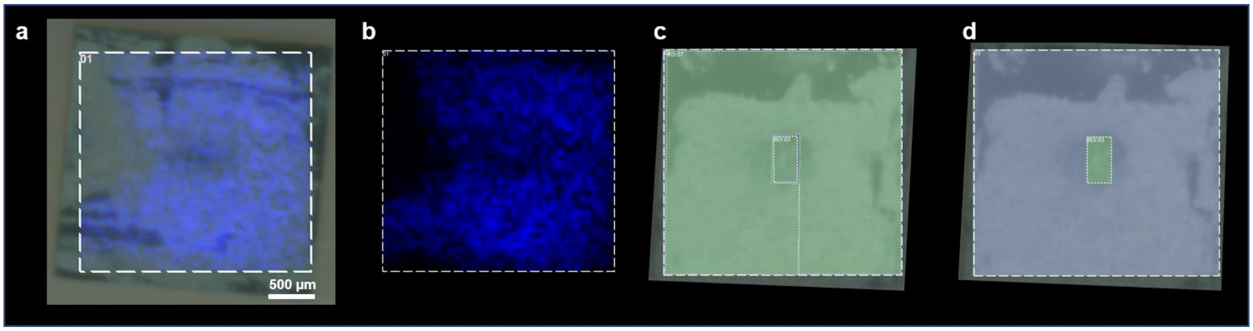

Supplementary Figure 3. **Chip region of interest (ROI) selection**. **a,** Optical image of chip and MALDI-IMS 2D map overlay for KLDL peptide. Imaged region defined by dashed white lines. **b,** MALDI-IMS 2D map only. **c,** Whole chip ROI selection for MALDI-IMS spectral averaging, where area is defined in green. d, Window ROI selection for MALDI-IMS spectral averaging, where window area is defined in green.

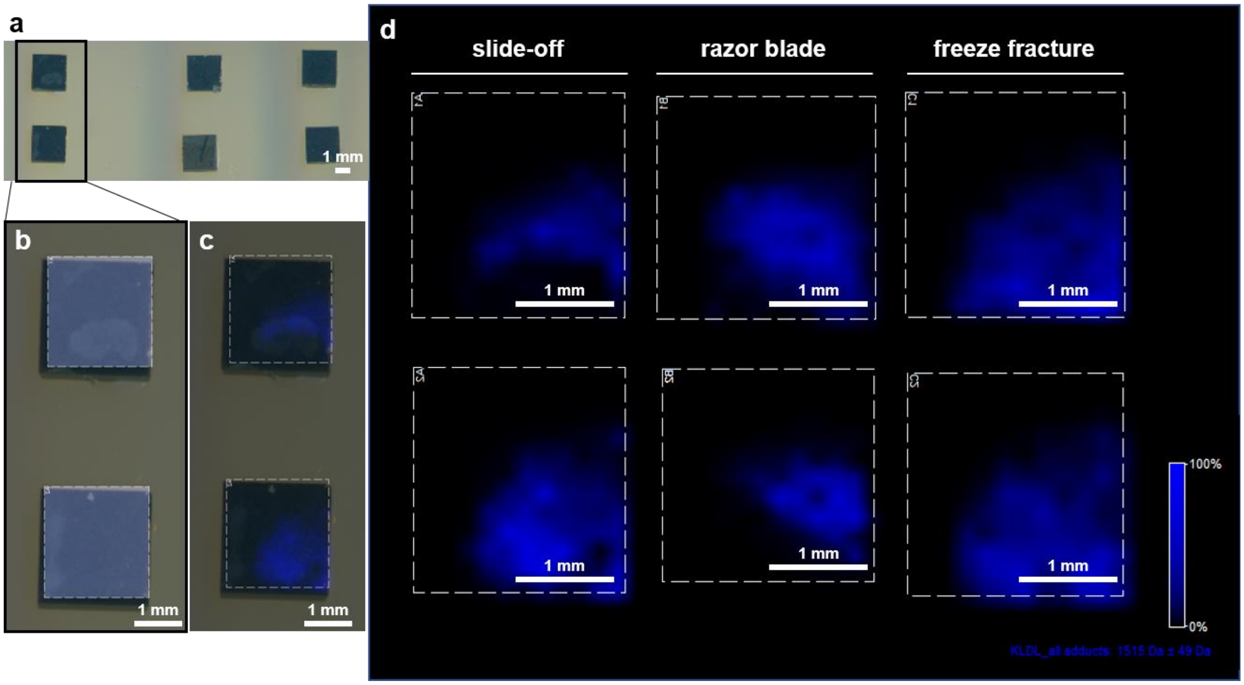

Supplementary Figure 4. Chip separation and distribution of material. Separation results in asymmetric distribution of material across surface, with material preferentially bound to one chip. **a,** Optical pictures of three sets of chips separated by the slide-off method (left), razor blade method (center), or freeze fracture method (right). **b,** Zoom-in of slide-off chips with MALDI-IMS measurement regions denoted by white box. **c,** Overlay of optical pictures and MALDI signals of chips shown in (b). **d,** MALDI signals only for the pairs of chips for each separation method. Filter (with manually defined range represented as ±) for KLDL $[M+Na^+]^+$ at 1493 ± 4.5 *m/z* is shown in blue. Filter colors are displayed as 0-100% of integrated intensity on a logarithmic scale within the analyzed region.

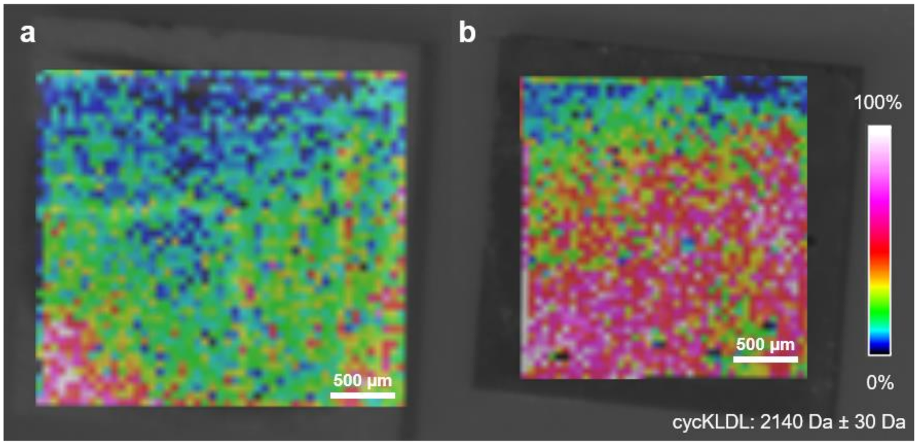

Supplementary Figure 5. Chip surface acidification. Optical picture with MALDI-IMS mapping overlay of chip surfaces for cycKLDL reveal that *post-mortem* treatment with 1% TFA does not change peptide distribution on chip surface after sample was initially dried. Notably, the same level of asymmetric sample distribution is detected under both conditions, which results from the initial chip separation after an LCTEM experiment. The significant difference, however, is that total MALDI-IMS intensities are uniformly higher when given this acid treatment. **a,** Optical picture with MALDI-IMS mapping overlay of chip surfaces for cycKLDL peptide that was imaged under low flux, pulsed conditions for 30 min. Chip was not treated with 1% TFA prior to matrix application. **b,** Optical picture with MALDI-IMS mapping overlay of chip surfaces for cycKLDL peptide that was imaged under low flux, pulsed conditions for 30 min. Chip was treated with 1% TFA prior to matrix application. Filter colors are displayed as 0-100% of integrated intensity on a logarithmic scale within the analyzed region.

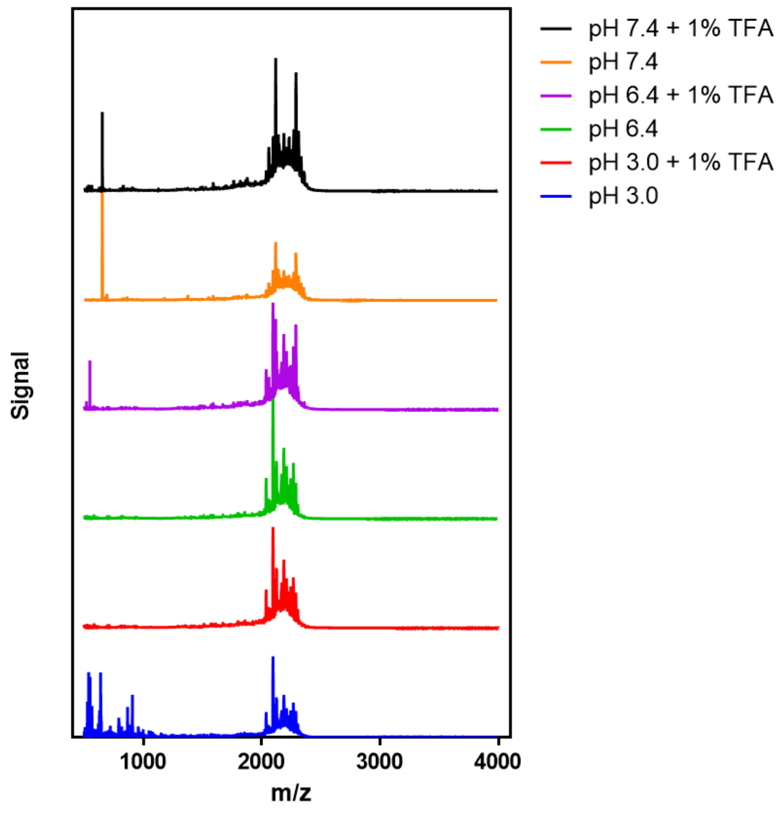

Supplementary Figure 6. Effect of pH and TFA on raw MALDI signal. Peptides dissolved at pH 7.4, 6.4, and 3.0 were deposited (1 µL) onto ITO slides for MALDI analysis. Spots were dried. For half of the spots, solutions of 1% TFA in water were deposited (1 µL) onto dry peptide spots and allowed to dry. Optimal signal was obtained at pH 6.4 with 1% TFA. Source data are provided as a Source Data file.

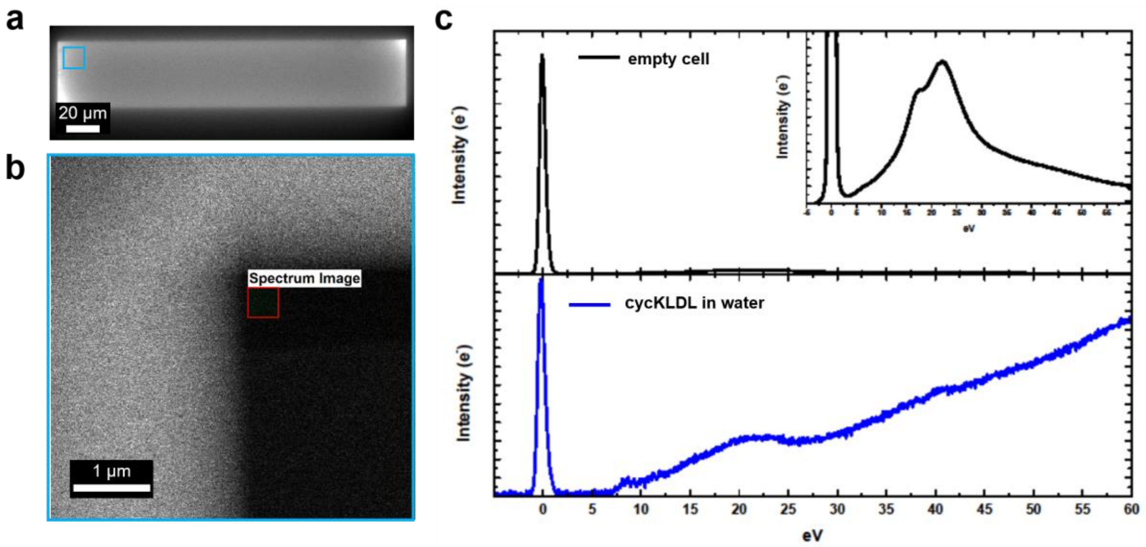

Supplementary Figure 7. EELS spectra of liquid cells to determine liquid layer thickness. Liquid cells contain cycKLDL peptide solution at 0.5 mg mL^{-1}. **a,** Low-magnification TEM image depicting a typical liquid cell used in this work. **b,** ADF image of one of the four corners of the cell. Red box indicates the spectrum image area where EELS data was collected. **c,** Corresponding EELS low-loss spectra from both empty and filled with cycKLDL in water cells. Inset shows magnified spectrum from empty cell, emphasizing the plasmon region. Source data are provided as a Source Data file. Each spectrum presented as average ± SD (n = 4 corners).

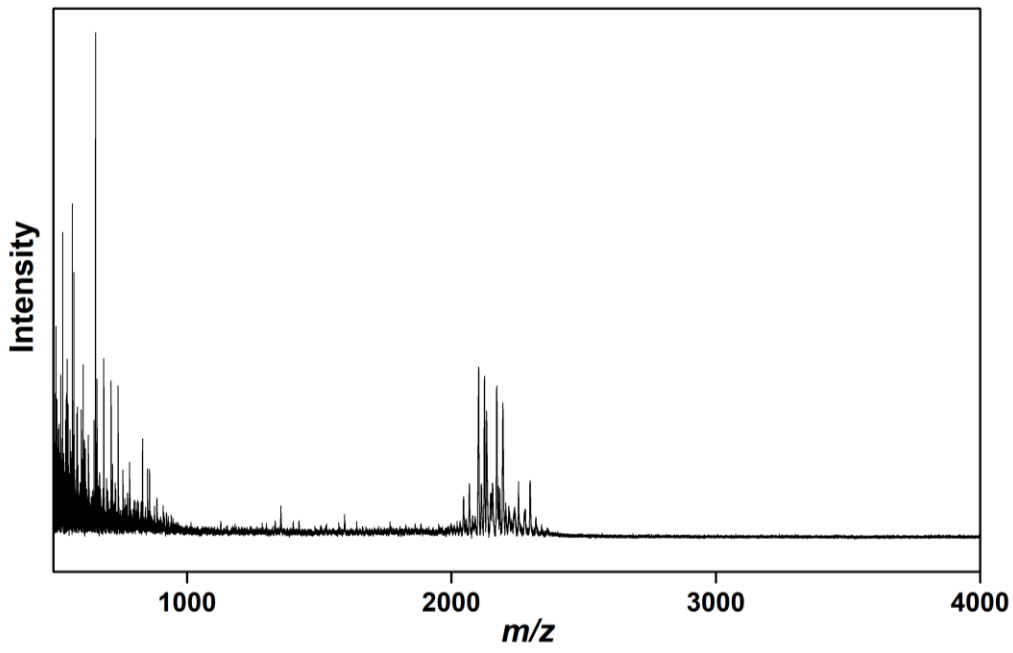

Supplementary Figure 8. Representative spectrum where matrix signal was high. Complete MALDI mass spectrum of cycKLDL peptide solution spotted and dried on a SiN_x chip, then sprayed with 8 layers of HCCA matrix containing 5% TFA, from 500-4000 *m/z*. High background matrix signal was detected from 500 *m/z* to approximately 1500 *m/z*. Source data are provided as a Source Data file.

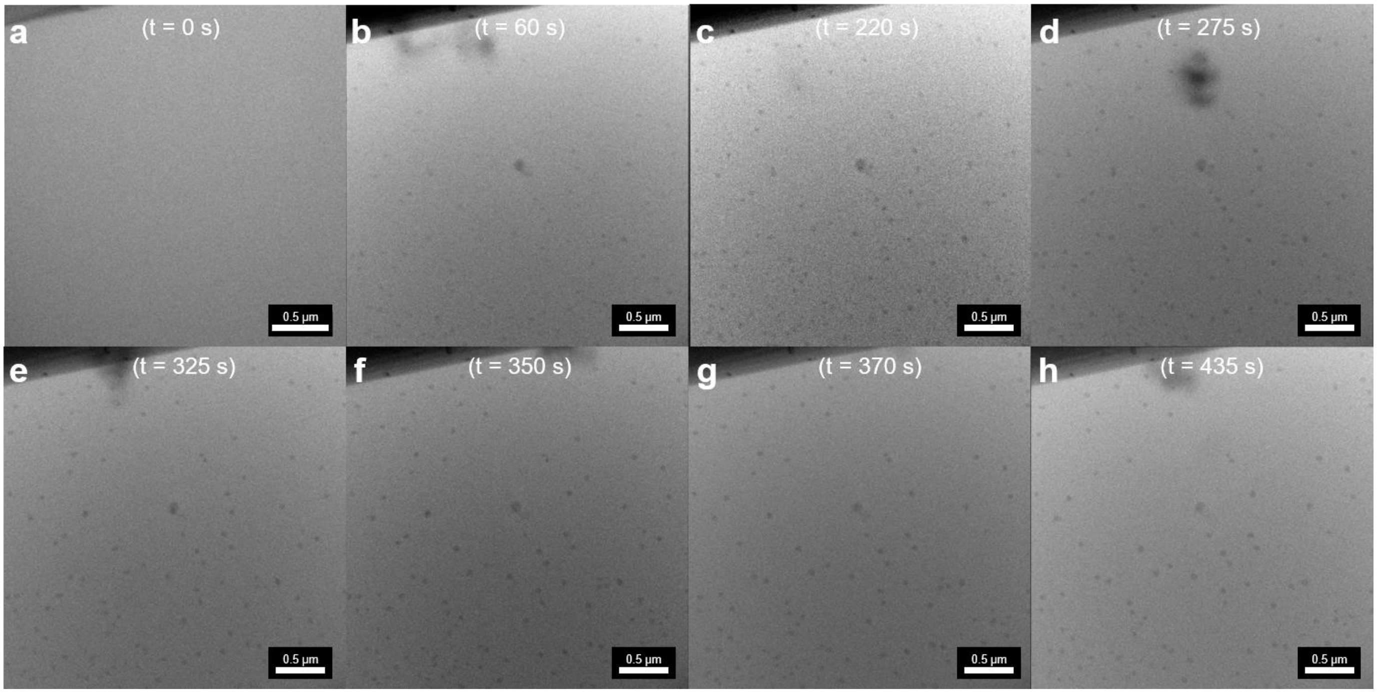

Supplementary Figure 9. **Full sequence of LCTEM images of cycKLDL under high flux conditions.** Gradual assembly of particle structures. **a-h,** Sequence of images acquired during imaging, with an appearance of large, high contrast structures at 60 s and on. Electron flux = 20.4 $e^-/Å^2s$, cumulative flux = 36,720 e^- $Å^{-2}$ s^{-1}.

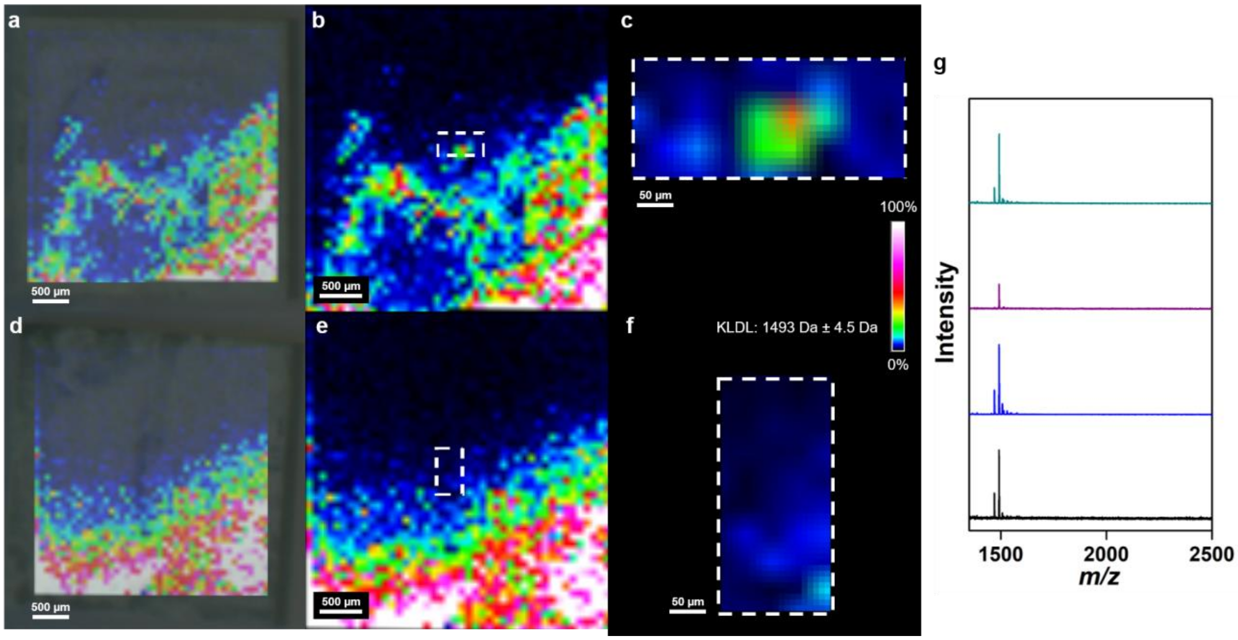

Supplementary Figure 10. MALDI-IMS of chips with KLDL after low flux, pulsed conditions for 30 min. **a**, Optical image of chip 1 with MALDI-IMS 2D map overlay. Filter (with manually defined range represented as ±) for KLDL $[M^{+}Na^{+}]^{+}$ at 1493 ± 4.5 *m/z* is applied. **b**, MALDI-IMS 2D map overlay with window region outlined with dashed lines. **c**, Zoom-in of MALDI-IMS map of window region shown in (**b**). **d**, Optical image of chip 2 with MALDI-IMS 2D map overlay. **e**, MALDI-IMS 2D map overlay with window region outlined with dashed lines. **f**, Zoom-in of MALDI-IMS map of window region shown in (**e**). **g**, MALDI mass spectra of the window of chip 1 (black), entire surface of chip 1 besides the window (blue), window of chip 2 (purple), entire surface of chip 2 besides the window (teal). Filter colors are displayed as 0-100% of integrated intensity on a logarithmic scale within the analyzed region. Source data are provided as a Source Data file.

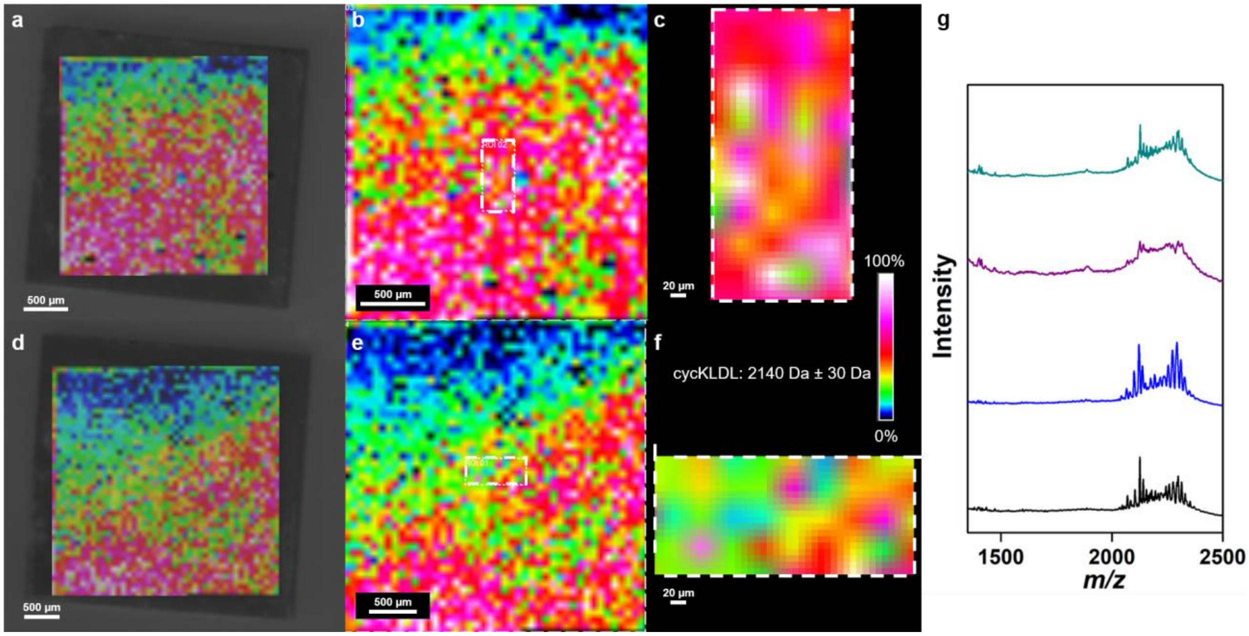

Supplementary Figure 11. MALDI-IMS of chips with cycKLDL after low flux, pulsed conditions for 30 min. **a**, Optical image of chip 1 with MALDI-IMS 2D map overlay. Filter (with manually defined range represented as ±) for cycKLDL $[M+H^{+}]^{+}$ at 2140 ± 30 *m/z* is applied. **b**, MALDI-IMS 2D map overlay with window region outlined with dashed lines. **c**, Zoom-in of MALDI-IMS map of window region shown in (**b**). **d**, Optical image of chip 2 with MALDI-IMS 2D map overlay. **e**, MALDI-IMS 2D map overlay with window region outlined with dashed lines. **f**, Zoom-in of MALDI-IMS map of window region shown in (**e**). **g**, MALDI mass spectra for window region of chip 1 (black), entire surface of chip 1 except the window region (blue), window region of chip 2 (purple), and entire surface of chip2 except the window region (teal). Filter colors are displayed as 0-100% of integrated intensity on a logarithmic scale within the analyzed region. Source data are provided as a Source Data file.

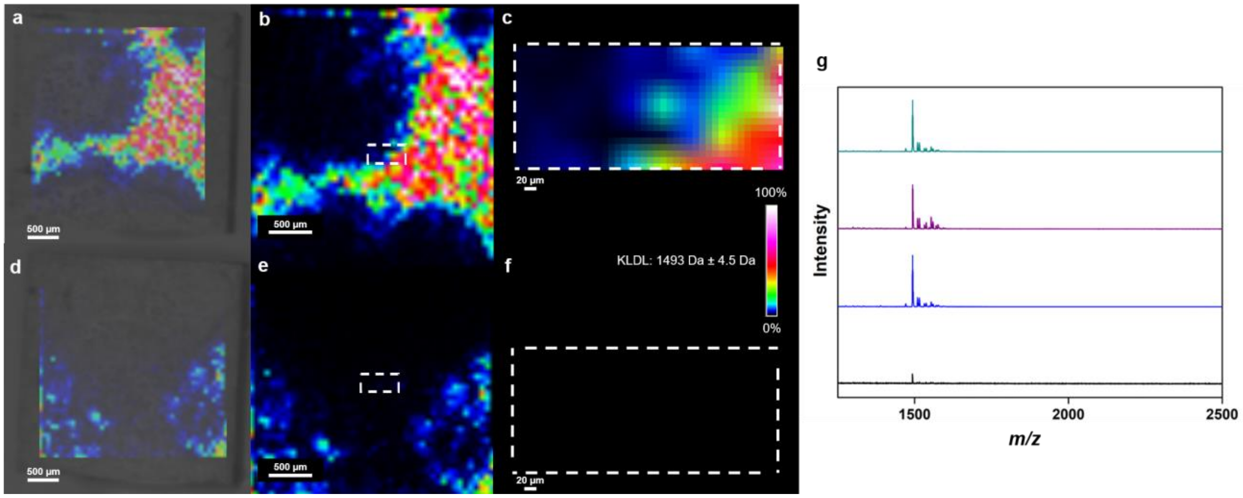

Supplementary Figure 12. MALDI-IMS of chips with KLDL after low flux conditions for 10 min. **a**, Optical image of chip 1 with MALDI-IMS 2D map overlay. Filter (with manually defined range represented as ±) for KLDL $[M^{+}Na^{+}]^{+}$ at 1493 ± 4.5 *m/z* is applied. **b**, MALDI-IMS 2D map overlay with window region outlined with dashed lines. **c**, Zoom-in of MALDI-IMS map of window region shown in (**b**). **d**, Optical image of chip 2 with MALDI-IMS 2D map overlay. **e**, MALDI-IMS 2D map overlay with window region outlined with dashed lines. **f**, Zoom-in of MALDI-IMS map of window region shown in (**e**). **g**, MALDI mass spectra of the window of chip 1 (black), entire surface of chip 1 besides the window (blue), window of chip 2 (purple), entire surface of chip 2 besides the window (teal). Filter colors are displayed as 0-100% of integrated intensity on a logarithmic scale within the analyzed region. Source data are provided as a Source Data file.

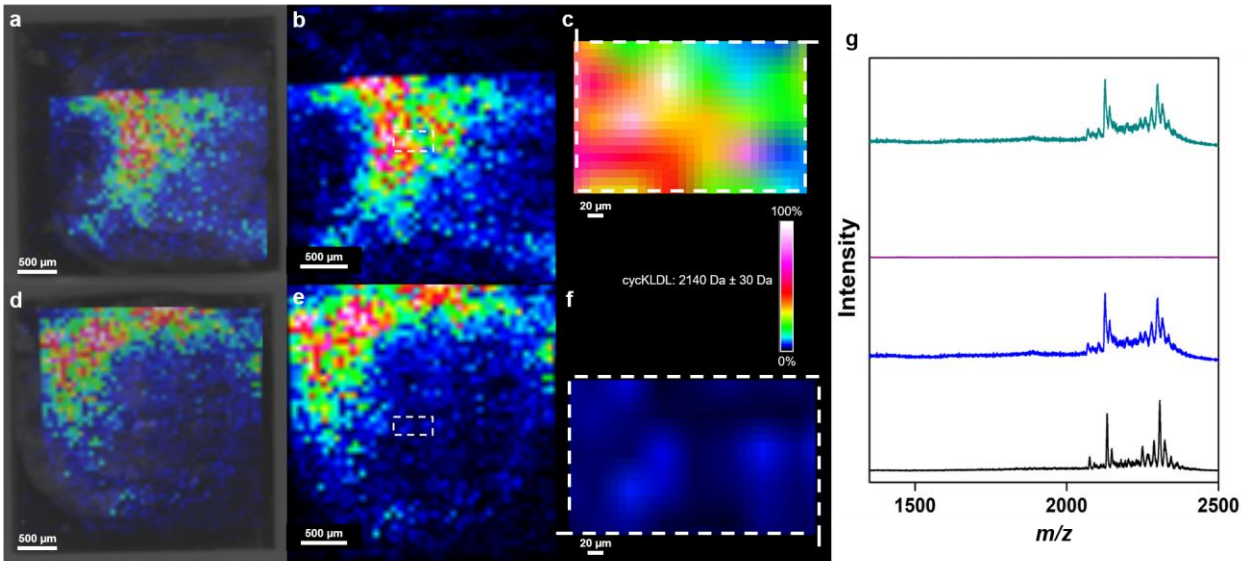

Supplementary Figure 13. MALDI-IMS of chips with cycKLDL after low flux conditions for 10 min. **a**, Optical image of chip 1 with MALDI-IMS 2D map overlay. Filter (with manually defined range represented as ±) for cycKLDL $[M+H^+]^+$ at 2140 ± 30 *m/z* is applied. **b**, MALDI-IMS 2D map overlay with window region outlined with dashed lines. **c**, Zoom-in of MALDI-IMS map of window region shown in (**b**). **d**, Optical image of chip 2 with MALDI-IMS 2D map overlay. **e**, MALDI-IMS 2D map overlay with window region outlined with dashed lines. **f**, Zoom-in of MALDI-IMS map of window region shown in (**e**). **g**, MALDI mass spectra for window region of chip 1 (black), entire surface of chip 1 except the window region (blue), window region of chip 2 (purple), and entire surface of chip2 except the window region (teal). Filter colors are displayed as 0-100% of integrated intensity on a logarithmic scale within the analyzed region. Source data are provided as a Source Data file.

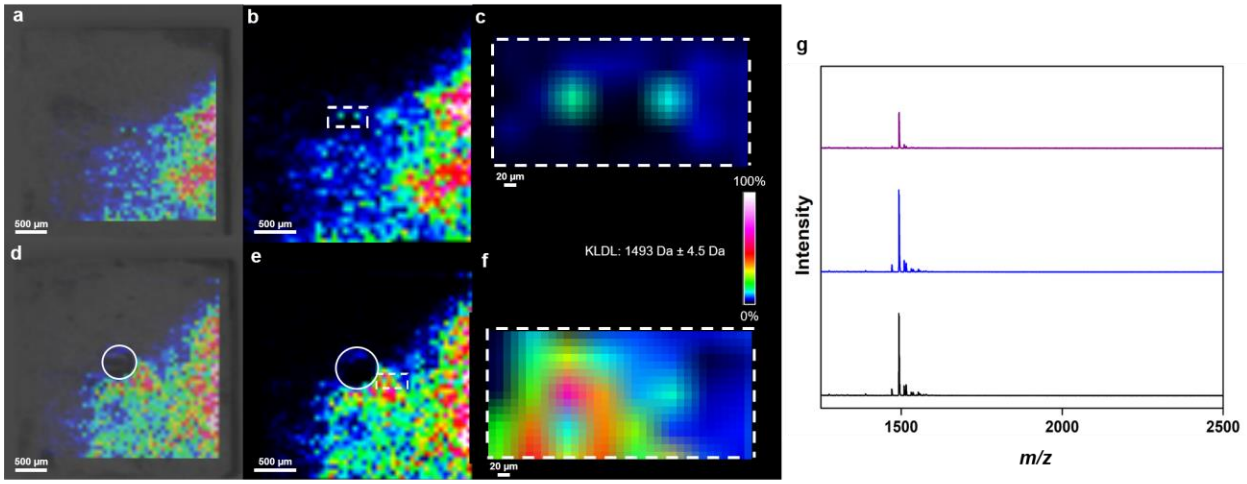

Supplementary Figure 14. MALDI-IMS of chips with KLDL after low flux conditions for 30 min. **a**, Optical image of chip 1 with MALDI-IMS 2D map overlay. Filter (with manually defined range represented as ±) for KLDL $[M^{+}Na^{+}]^{+}$ at 1493 ± 4.5 *m/z* is applied. **b**, MALDI-IMS 2D map overlay with window region outlined with dashed lines. **c**, Zoom-in of MALDI-IMS map of window region shown in (**b**). **d**, Optical image of chip 2 with MALDI-IMS 2D map overlay. Window of chip had broken while mounting onto ITO slide and glue seeped through onto surface, removing any peptide signal. This glue region is circled in white. **e**, MALDI-IMS 2D map overlay with window region outlined with dashed lines. **f**, Zoom-in of MALDI-IMS map of region shown in (**e**), just off to the side of the glue spot. **g**, MALDI mass spectra for the surface of chip 1 except the window region (black), entire surface of chip 2 except the region outlined in a circle (blue), and window region of chip 1 (purple). Filter colors are displayed as 0-100% of integrated intensity on a logarithmic scale within the analyzed region. Source data are provided as a Source Data file.

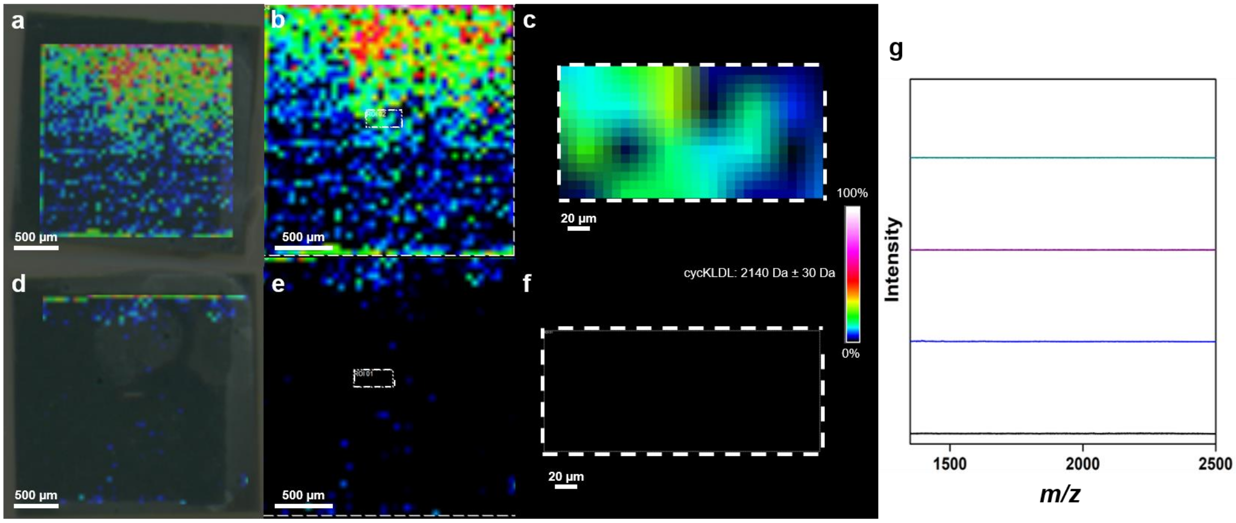

Supplementary Figure 15. MALDI-IMS of chips with cycKLDL after low flux conditions for 30 min. **a**, Optical image of chip 1 with MALDI-IMS 2D map overlay. Filter (with manually defined range represented as ±) for cycKLDL $[M+H^+]^+$ at 2140 ± 30 *m/z* is applied. **b**, MALDI-IMS 2D map overlay with window region outlined with dashed lines. **c**, Zoom-in of MALDI-IMS map of window region shown in (**b**). **d**, Optical image of chip 2 with MALDI-IMS 2D map overlay. **e**, MALDI-IMS 2D map overlay with window region outlined with dashed lines. **f**, Zoom-in of MALDI-IMS map of window region shown in (**e**). **g**, MALDI mass spectra for the window region of chip 1 (black), entire surface of chip 1 except the window region (blue), window region of chip 2 (purple), and entire surface of chip 2 except window region (teal). Filter colors are displayed as 0-100% of integrated intensity on a logarithmic scale within the analyzed region. Source data are provided as a Source Data file.

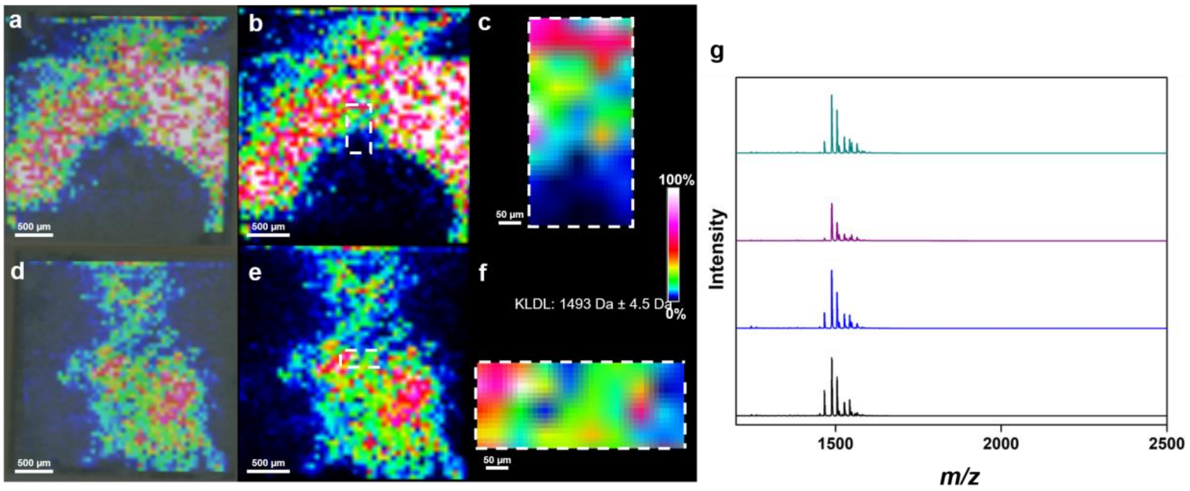

Supplementary Figure 16. MALDI-IMS of chips with KLDL after high flux, pulsed conditions for 30 min. **a**, Optical image of chip 1 with MALDI-IMS 2D map overlay. Filter (with manually defined range represented as ±) for KLDL $[M^{+}Na^{+}]^{+}$ at 1493 ± 4.5 *m/z* is applied. **b**, MALDI-IMS 2D map overlay with window region outlined with dashed lines. **c**, Zoom-in of MALDI-IMS map of window region shown in (**b**). **d**, Optical image of chip 2 with MALDI-IMS 2D map overlay. **e**, MALDI-IMS 2D map overlay with window region outlined with dashed lines. **f**, Zoom-in of MALDI-IMS map of window region shown in (**e**). **g**, MALDI mass spectra of the window of chip 1 (black), entire surface of chip 1 besides the window (blue), window of chip 2 (purple), entire surface of chip 2 besides the window (teal). Filter colors are displayed as 0-100% of integrated intensity on a logarithmic scale within the analyzed region. Source data are provided as a Source Data file.

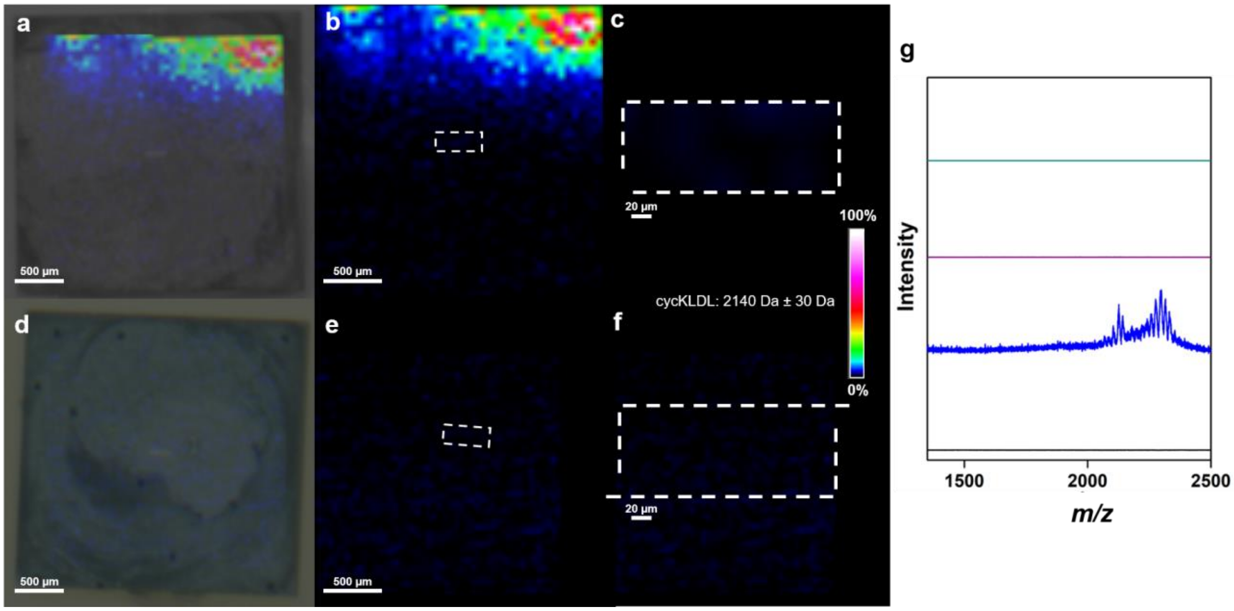

Supplementary Figure 17. MALDI-IMS of chips with cycKLDL after high flux, pulsed conditions for 30 min. **a**, Optical image of chip 1 with MALDI-IMS 2D map overlay. Filter (with manually defined range represented as ±) for cycKLDL $[M+H^+]^+$ at 2140 ± 30 *m/z* is applied. **b**, MALDI-IMS 2D map overlay with window region outlined with dashed lines. **c**, Zoom-in of MALDI-IMS map of window region shown in (**b**). **d**, Optical image of chip 2 with MALDI-IMS 2D map overlay. **e**, MALDI-IMS 2D map overlay with window region outlined with dashed lines. **f**, Zoom-in of MALDI-IMS map of window region shown in (**e**). **g**, MALDI mass spectra for the window region of chip 1 (black), entire surface of chip 1 except the window region (blue), window region of chip 2 (purple), and entire surface of chip 2 except window region (teal). Filter colors are displayed as 0-100% of integrated intensity on a logarithmic scale within the analyzed region. Source data are provided as a Source Data file.

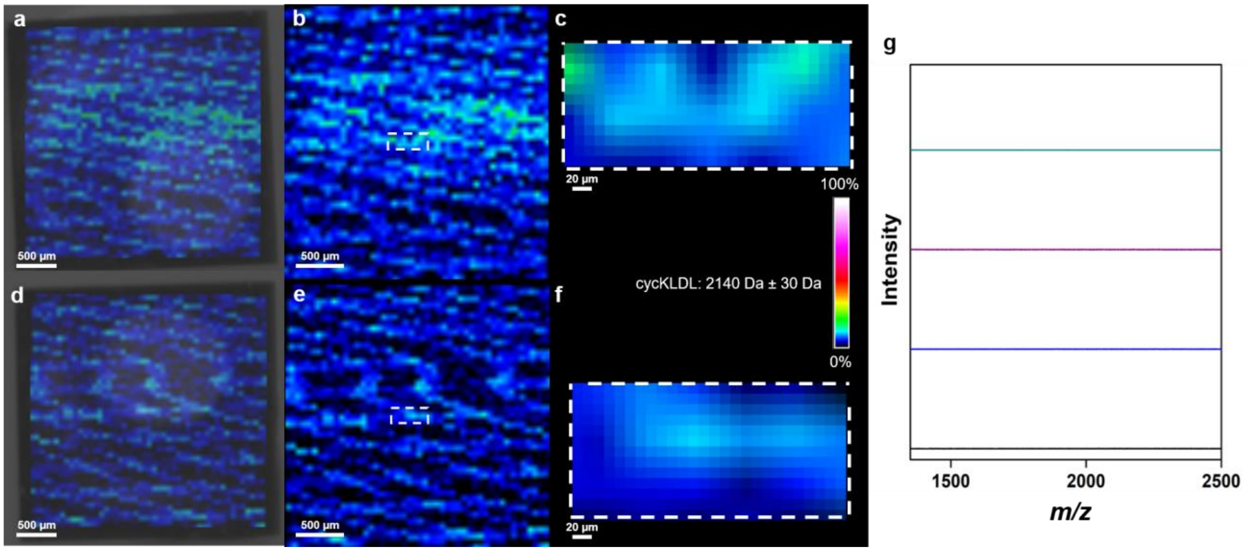

Supplementary Figure 18. MALDI-IMS of chips with cycKLDL after high flux conditions for 10 min. **a**, Optical image of chip 1 with MALDI-IMS 2D map overlay. Filter (with manually defined range represented as ±) for cycKLDL $[M+H^+]^+$ at 2140 ± 30 *m/z* is applied. **b**, MALDI-IMS 2D map overlay with window region outlined with dashed lines. **c**, Zoom-in of MALDI-IMS map of window region shown in (**b**). **d**, Optical image of chip 2 with MALDI-IMS 2D map overlay. **e**, MALDI-IMS 2D map overlay with window region outlined with dashed lines. **f**, Zoom-in of MALDI-IMS map of window region shown in (**e**). **g**, MALDI mass spectra for the window region of chip 1 (black), entire surface of chip 1 except the window region (blue), window region of chip 2 (purple), and entire surface of chip 2 except window region (teal). Filter colors are displayed as 0-100% of integrated intensity on a logarithmic scale within the analyzed region. Source data are provided as a Source Data file.

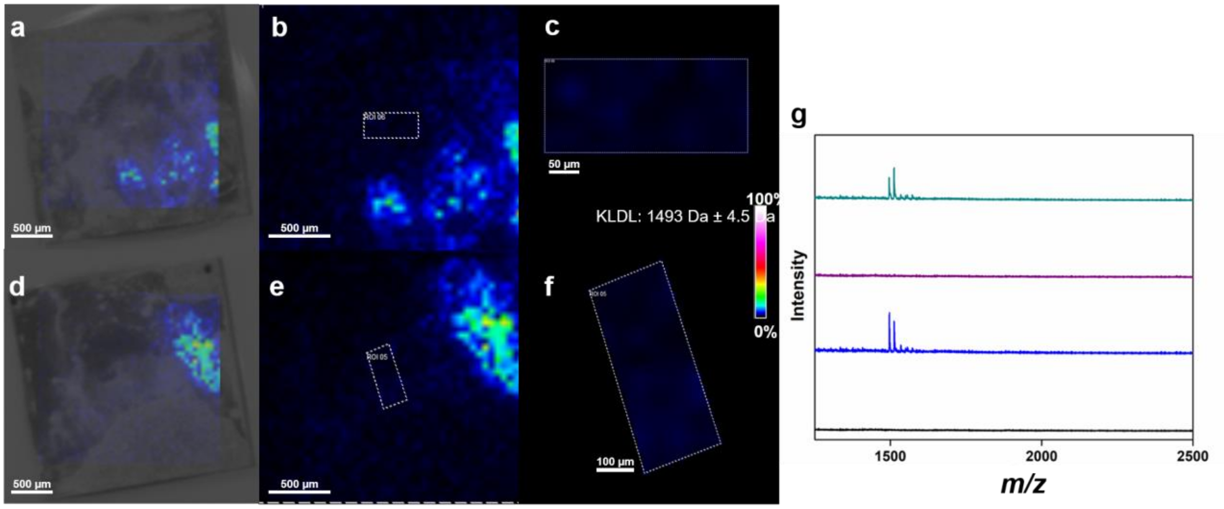

Supplementary Figure 19. MALDI-IMS of chips with KLDL after high flux conditions for 30 min. **a**, Optical image of chip 1 with MALDI-IMS 2D map overlay. Filter (with manually defined range represented as ±) for KLDL $[M^{+}Na^{+}]^{+}$ at 1493 ± 4.5 *m/z* is applied. **b**, MALDI-IMS 2D map overlay with window region outlined with dashed lines. **c**, Zoom-in of MALDI-IMS map of window region shown in (**b**). **d**, Optical image of chip 2 with MALDI-IMS 2D map overlay. **e**, MALDI-IMS 2D map overlay with window region outlined with dashed lines. **f**, Zoom-in of MALDI-IMS map of window region shown in (**e**). **g**, MALDI mass spectra of the window of chip 1 (black), entire surface of chip 1 besides the window (blue), window of chip 2 (purple), entire surface of chip 2 besides the window (teal). Filter colors are displayed as 0-100% of integrated intensity on a logarithmic scale within the analyzed region. Source data are provided as a Source Data file.

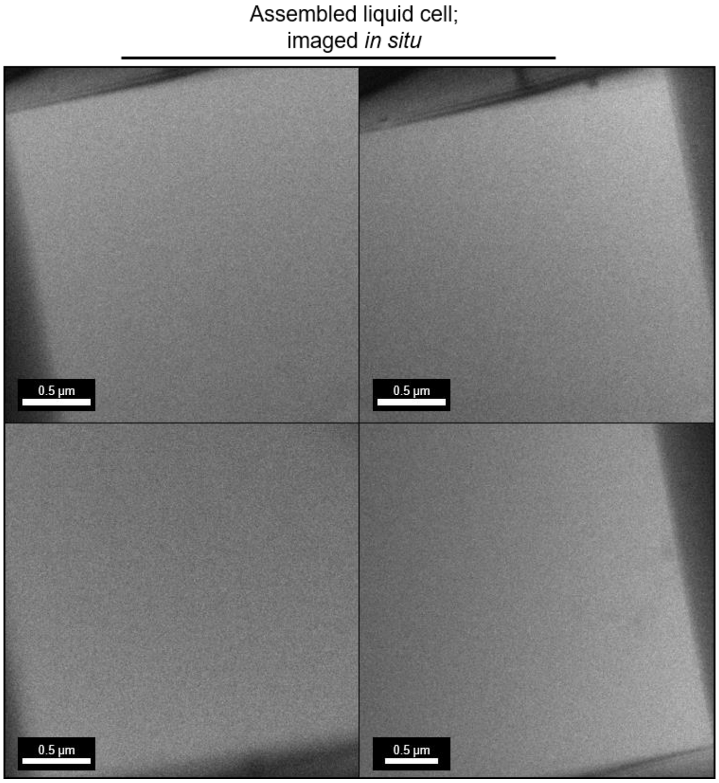

Supplementary Figure 20. cycKLDL + inactive TCEP control. Images of each corner of a liquid cell containing cycKLDL and inactive TCEP (pH 3.0), which had been imaged *in situ* under low flux, pulsed conditions, acquired 1 hr after initial mixing of cycKLDL and TCEP outside of the liquid cell. No structure formation was observed. The material with minimal contrast in the bottom right corner of the window (lower right panel) was present throughout imaging and was most likely an artifact of liquid cell assembly. Electron flux = 0.98 e^{-} $Å^{-2}$ s^{-1}.

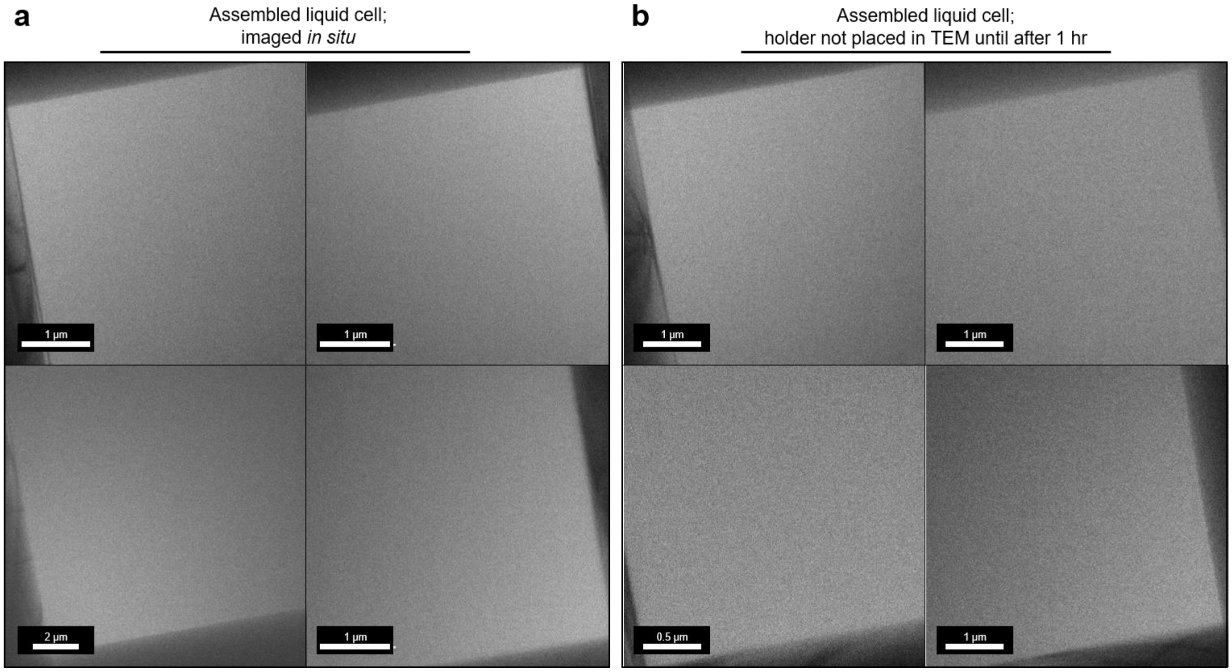

Supplementary Figure 21. cycKLDL + inactive thermolysin controls. a, Images of each corner of a liquid cell containing cycKLDL and inactive thermolysin, which had been imaged *in situ* under low flux, pulsed conditions, acquired 1 hr after initial mixing of cycKLDL and inactive thermolysin outside of the liquid cell. No structure formation was observed. Electron flux = 0.41 e^{-} $Å^{-2}$ s^{-1}. **b**, Images of each corner of a liquid cell containing cycKLDL and inactive thermolysin after the liquid cell containing cycKLDL and inactive thermolysin remained on a benchtop for 60 min, as a no flux control. No structures could be resolved.

#	Imaging Conditions	Average Flux [$e^- Å^{-2} s^{-1}$]	Cumulative Flux [$e^- Å^{-2}$]	KLDL: Structure formation observed by LCTEM	KLDL: MALDI-IMS signal detected	cycKLDL: Structure formation observed by LCTEM	cycKLDL: MALDI-IMS signal detected
1	No flux	N/A	N/A	N/A	N/A	N/A	N/A
2	Low flux, pulsed, 30 min total	0.11 ± 0.07	3.3	N	Y	N	Y
3	High flux, pulsed, 30 min total	27.8 ± 5.7	840	N	Y	N	N
4	Low flux, 10 min continuous	0.11 ± 0.07	66	N	Y	N	Y
5	Low flux, 30 min continuous	0.11 ± 0.07	200	N	Y	N	N
6	Low flux, 60 min continuous	0.11 ± 0.07	400	N	N	N	N
7	High flux, 10 min continuous	27.8 ± 5.7	16,700	N	N	N	N
8	High flux, 30 min continuous	27.8 ± 5.7	50,100	Y	N	Y	N

Supplementary Table 1. LCTEM imaging conditions utilized to examine beam-induced damage. KLDL and cycKLDL under various flux and cumulative flux conditions. Average flux is mean ± SD (n = 3 repeats).
